# Low-intensity pulsed ultrasound promotes the osteogenesis of mechanical force-treated periodontal ligament cells via Piezo1

**DOI:** 10.3389/fbioe.2024.1347406

**Published:** 2024-04-17

**Authors:** Fu Zheng, Tong Wu, Feifei Wang, Huazhi Li, Hongyi Tang, Xinyu Cui, Cuiying Li, Yixiang Wang, Jiuhui Jiang

**Affiliations:** ^1^ Department of Orthodontics, Peking University School and Hospital of Stomatology, Haidian, Beijing, China; ^2^ National Clinical Research Center for Oral Diseases and National Engineering Laboratory for Digital and Material Technology of Stomatology, Haidian, Beijing, China; ^3^ Beijing Key Laboratory of Digital Stomatology, Haidian, Beijing, China; ^4^ Center of Digital Dentistry, Peking University School and Hospital of Stomatology, Haidian, Beijing, China; ^5^ Central Laboratory, Peking University School and Hospital of Stomatology, Haidian, Beijing, China

**Keywords:** low-intensity pulsed ultrasound, orthodontic tooth movement, periodontal ligament cells, osteogenesis, Piezo1

## Abstract

**Background:**

Low-intensity pulsed ultrasound (LIPUS) can accelerate tooth movement and preserve tooth and bone integrity during orthodontic treatment. However, the mechanisms by which LIPUS affects tissue remodeling during orthodontic tooth movement (OTM) remain unclear. Periodontal ligament cells (PDLCs) are pivotal in maintaining periodontal tissue equilibrium when subjected to mechanical stimuli. One notable mechano-sensitive ion channel, Piezo1, can modulate cellular function in response to mechanical cues. This study aimed to elucidate the involvement of Piezo1 in the osteogenic response of force-treated PDLCs when stimulated by LIPUS.

**Method:**

After establishing rat OTM models, LIPUS was used to stimulate rats locally. OTM distance and alveolar bone density were assessed using micro-computed tomography, and histological analyses included hematoxylin and eosin staining, tartrate-resistant acid phosphatase staining and immunohistochemical staining. GsMTx4 and Yoda1 were respectively utilized for Piezo1 functional inhibition and activation experiments in rats. We isolated human PDLCs (hPDLCs) *in vitro* and evaluated the effects of LIPUS on the osteogenic differentiation of force-treated hPDLCs using real-time quantitative PCR, Western blot, alkaline phosphatase and alizarin red staining. Small interfering RNA and Yoda1 were employed to validate the role of Piezo1 in this process.

**Results:**

LIPUS promoted osteoclast differentiation and accelerated OTM in rats. Furthermore, LIPUS alleviated alveolar bone resorption under pressure and enhanced osteogenesis of force-treated PDLCs both *in vivo* and *in vitro* by downregulating Piezo1 expression. Subsequent administration of GsMTx4 in rats and siPIEZO1 transfection in hPDLCs attenuated the inhibitory effect on osteogenic differentiation under pressure, whereas LIPUS efficacy was partially mitigated. Yoda1 treatment inhibited osteogenic differentiation of hPDLCs, resulting in reduced expression of Collagen Ⅰα1 and osteocalcin in the periodontal ligament. However, LIPUS administration was able to counteract these effects.

**Conclusion:**

This research unveils that LIPUS promotes the osteogenesis of force-treated PDLCs via downregulating Piezo1.

## 1 Introduction

Orthodontic treatments typically require a duration of 20 months or even longer (El-Angbawi et al., 2023). Long-term orthodontic treatment may lead to reduced patient compliance and some complications (El-Bialy et al., 2020; Al-Dboush et al., 2021; El-Angbawi et al., 2023). Low intensity pulsed ultrasound (LIPUS) is seen as a method to accelerate orthodontic tooth movement (OTM). Through split-mouth controlled trials, El Bialy et al. found that LIPUS can accelerate OTM and reduce treatment duration, as well as minimize orthodontically induced root resorption, regardless of whether fixed or invisible orthodontic treatment is used (Kaur and El-Bialy, 2020; Al-Dboush et al., 2021). However, some scholars argue that LIPUS does not accelerate OTM (Al-Daghreer et al., 2014; Qamruddin et al., 2021). The controversy surrounding the effectiveness of LIPUS in OTM arises from the unclear understanding of its mechanism of action on orthodontic bone remodeling, necessitating further investigation. Osteoclast differentiation serves as the initiating factor for initiating OTM (Han et al., 2022; Xie et al., 2022). Therefore, the assessment of the impact of LIPUS on the rate OTM mainly relies on its potential influence on osteoclast differentiation on the pressure side.

Furthermore, long-term orthodontic treatment induced alveolar bone resorption and periodontal supporting tissue destruction are caused by upregulated osteoclast activity and downregulated tissue repair capacity under pressure (Feng et al., 2016; Wang et al., 2018; Zhou et al., 2023). Therefore, the balance between tissue destruction and repair during orthodontic treatment is crucial for maintaining periodontal health (Meikle, 2005; Li et al., 2021). The periodontal ligament cells (PDLCs) comprise multiple cell types, including periodontal ligament stem cells (PDLSCs) and periodontal ligament fibroblasts (PDLFs), which are essential for maintaining periodontal tissue homeostasis during OTM (Seo et al., 2004; Wang et al., 2018; Jin et al., 2020; Jiang et al., 2021; Jiang et al., 2024). Orthodontic researchers typically employ compressive force to simulate the pressure experienced on the compression side of teeth during OTM when culturing PDLCs *in vitro*. It has been demonstrated that static pressure stimulation inhibits the osteogenic differentiation of PDLCs (Feng et al., 2016; Zhang et al., 2016). LIPUS activates the PI3K/AKT signaling pathway and enhances the proliferation of human bone marrow mesenchymal stem cells (Xie et al., 2019). After LIPUS stimulation, human PDLSCs (hPDLSCs) show a stronger ability to form cell sheets and differentiate into osteogenic cells (Li et al., 2020b). LIPUS facilitates the osteogenesis of PDLSCs isolated from patients who are troubled by periodontitis via the unfolded protein response pathway (Li et al., 2020a). Nevertheless, there is almost no research on the impact of LIPUS on mechanical force-treated PDLCs. Thus, further investigation is needed to elucidate the effects of LIPUS on the osteogenic differentiation ability of PDLCs subsequent to mechanical force stimulation, with the aim of elucidating its potential mechanism for maintaining periodontal homeostasis during OTM.

For LIPUS to exert its effects, the process requires cells to sense mechanical stimuli and convert mechanical signals into biological signals, thereby triggering downstream biological effects. Piezo1 is a transmembrane ion channel protein that responds to mechanical stimuli and generates voltage-dependent ion channel activity, thus playing a crucial role in mammalian physiological processes (Coste et al., 2010). Piezo1 is crucial for bone development and bone homeostasis related to mechanical stimulation (Wang et al., 2020; Qin et al., 2021). It has been proven that Piezo1 contributes to periodontium remodeling in response to orthodontic force. Tensile force activates Piezo1 of periodontal ligament cells, thereby promoting alveolar bone remodeling on the tension side (Jiang et al., 2021; Du and Yang, 2023). Additionally, compressive loading upregulates the ratio of the receptor activator of NF-κB ligand to osteoprotegerin (RANKL/OPG) in periodontal ligament fibroblasts through Piezo1, subsequently promoting osteoclast differentiation (Schröder et al., 2023). Furthermore, Piezo1 responds to the mechanical signals of LIPUS and converts them into intracellular calcium ion signals, thereby promoting the proliferation of MC3T3-E1 and dental pulp stem cells (Gao et al., 2017; Zhang et al., 2021). LIPUS also enhances the expression of Piezo1 and facilitates the endothelial differentiation and microvascular formation of PDLSCs (Hu et al., 2022). Nevertheless, whether and how Piezo1 functions to induce the effects of LIPUS on the osteogenesis of mechanical force-treated PDLCs remain inadequately understood.

Therefore, in this study, we established a rat OTM model *in vivo* and examined isolated hPDLSCs *in vitro* to investigate the effects of mechanical loading. On this basis, we used LIPUS to stimulate rats and hPDLSCs. We aimed to explore the following issues in this study: 1) the effects of LIPUS on osteoclast differentiation on the pressure side and the rate of OTM; 2) the effects of LIPUS on the osteogenesis of force-treated PDLCs and the alveolar bone density on the pressure side; and 3) the role of Piezo1 in the osteogenic differentiation of force-treated PDLCs mediated by LIPUS.

## 2 Materials and methods

### 2.1 Ethic statement

All research protocols were approved by the Ethics Committee of Peking University School of Stomatology, Beijing, China (PKUSSIRB-202385020). We obtained informed consent from all donors and their legal guardians prior to collecting samples. All animal experiments conducted in this study followed standard procedures and adhered to ethical principles for animal welfare. The animal experimental protocols were approved by the Institutional Animal Care and Use Committee of Peking University (LA2022288).

### 2.2 Isolation, culture, and identification of human PDLCs

After being isolated from extracted teeth, human PDLCs (hPDLCs) were cultured according to the established protocols (Seo et al., 2004; Xie et al., 2022). These cells, obtained from 3 individuals aged 12–25 years, were combined and maintained in α-MEM (Gibco, Grand Island, NY, United States) supplemented with 15% fetal bovine serum (FBS; Gibco), 0.292 mg/mL of glutamine (Gibco), 100 U/mL of penicillin and 100 μg/mL of streptomycin (Solarbio, Tongzhou, Beijing, China) and incubated at 37°C under a humidified atmosphere containing 5% CO_2_.

Flow cytometry (Accuri C6, BD, Franklin Lakes, NJ, United States) was used to detect surface markers of cells, including CD31 (Anti-CD31, ab9498, Abcam, Boston, MA, United States), CD34 (Anti-CD34, ab8536, Abcam), CD45 (Anti-CD45, ab40763, Abcam), CD29 (Anti-CD29, ab30394, Abcam), CD90 (Anti-CD90, EPR28145-53, Abcam) and PE-CD105 (anti-CD105, 303205, Biolegend, San Diego, CA, United States) (Chang et al., 2022). FITC-conjugated rabbit IgG (ab6717, Abcam) and PE-conjugated rabbit IgG (ab7003, Abcam) were used as secondary antibodies.

Subsequent experiments utilized cells at passages 3–6.

### 2.3 Osteogenic induction of hPDLCs

hPDLCs (1 × 10^5^/mL) were seeded into 6/24-well plates and cultured. Until the cell confluence reached 70%–80%, the osteogenic-inducing medium containing 100 μM ascorbic acid (Sigma-Aldrich, St. Louis, MO, United States), 2 mM β-glycerophosphate (Sigma), and 10 nM dexamethasone (Sigma) (Zhang et al., 2022) were added and the medium was changed every 2 days. Alkaline Phosphatase (ALP) staining (Beyotime, Shanghai, China) was performed at 7 days after osteogenic induction, and Alizarin Red staining (Sigma) was performed at 21 days after osteogenic induction. According to our previous procedure (Zhan et al., 2020), 10% (w/v) cetyl-pyridinium chloride (CPC, HB11089, Macklin, Shanghai, China) was added to dissolve the red matrix sediment to detect the degree of mineralization quantitatively. The absorbance of the solution was measured at 490 nm OD using an ELx808™ plate reader (Bio Tek, Vermont, United States). The samples for quantitative real-time polymerase chain reaction (qPCR) and Western blot analyses were collected at 7 days after osteogenic induction. Each experiment was repeated three times.

### 2.4 Application of mechanical stress to hPDLCs

Static compressive force was applied to hPDLCs following a previously established method (Jin et al., 2020). In brief, a glass layer with added metal balls was placed on top of a cell layer cultured in 6/24-well plates, which had reached 70%–80% confluence (Supplementary Figure S1A). We exposed hPDLCs to static compressive force at 1 g/cm^2^ for a duration of 24 h (Wang et al., 2018; Jin et al., 2020). Then, we removed the loading device and proceeded with subsequent processing.

### 2.5 LIPUS stimulation to hPDLCs

The LIPUS exposure device manufactured by the Institute of Acoustics (Chinese Academy of Sciences, China) consisted of an array of 6 big transducers (each with a diameter of 34 mm, designed for use in a 6-well culture plate) and 6 small transducers (each with a diameter of 15 mm, designed for use in a 24-well culture plate and SD rats). The LIPUS conditions used in this study were consistent with those commonly used in previous studies, with a frequency of 1.5 MHz, a pulse duty cycle of 1:4, a pulse repetition frequency of 1.0 kHz, and an average intensity of 30 mW/cm^2^ (El-Bialy et al., 2020).

The *in vitro* experiments were carried out at 37°C, and LIPUS was applied to hPDLCs after static compressive force was removed for 20 min each day before harvesting. To carry out these experiments, the culture plates were placed on the ultrasound transducer with a thin layer of standard ultrasound gel. The sham controls were handled in the same way as the treated samples, but the ultrasound generator was not turned on (Supplementary Figure S1B–D).

### 2.6 Small interfering RNA transfection and Yoda1 stimulation of hPDLCs

The transfection experiments with small interfering RNA (siRNA) were conducted once the cells achieved 80% confluence, using a riboFECT™ CP Transfection Kit (RiboBio, Guangzhou, China) and following the manufacturer’s instructions. To knockdown Piezo1, hPDLSCs were transfected with siPIEZO1 (siG150327130626-1-5, RiboBio, Guangzhou, China). The transfection reagent mixture was added to the medium for 48 h prior to the compressive force stimulation.

According to the manufacturer’s instructions, Yoda1 (S6678, Selleck, Houston, TX, United States) was dissolved in dimethyl sulfoxide (DMSO) to prepare a stock solution of 50 mM. To activate Piezo1, hPDLCs were treated with the pharmacological agonist Yoda1 at a final concentration of 10 μM (diluted in the culture medium) for 24 h prior to osteogenic induction (Sugimoto et al., 2017). For the control group, an equal volume of DMSO was added.

### 2.7 Conditional medium and osteoclastic induction of RAW264.7

The RAW264.7 cell lines were purchased from Procell Life Science and Technology Co., Ltd. (Procell, Jiangxia, Wuhan, China). Cells were cultured in DMEM high glucose medium (Gibco) supplemented with 10% FBS (Gibco), and incubated at 37°C in a humidified atmosphere containing 5% CO_2_. Subsequent experiments utilized cells within passages 10.

The culture medium of hPDLCs in each group was collected after removing static pressure and applying 20 min of LIPUS stimulation. After centrifugation, the supernatant was retained and stored at −20°C. Raw264.7 cells (5 × 10^4^/mL) were seeded into 6/24-well plates and cultured overnight. And on the second day, the conditioned medium consisting of 50% supernatant, 50% DMEM and 10 ng/mL RANKL (R&D, Emeryville, MN, United States) were used to induce osteoclastic differentiation (Xin et al., 2020). The conditioned medium was changed every 3 days. After 5 days induction, the cells were collected for qPCR and TRAP staining. TRAP staining was performed using an acid phosphatase kit (387A-1KT; Sigma) according to the manufacturer’s instructions. TRAP-positive, multinucleated (≥3 nuclei) cells were counted in each well. Each experiment was repeated three times.

### 2.8 qPCR

As previously described (Chen et al., 2022a), we extracted RNA from hPDLCs and RAW264.7 cells using TRIzol reagent (Invitrogen, Carlsbad, CA, United States) as per the manufacturer’s guidelines. The purity of the isolated RNA was assessed by measuring the A260/A280 ratio using a spectrophotometer (Nanodrop 8000, Thermo, Waltham, MA, United States), and only samples with ratios between 1.8 and 2.0 were used for downstream experiments. RNA was reverse transcribed into complementary first-strand cDNA using cDNA synthesis kits (Takara Bio, Tokyo, Japan), followed by amplification using qPCR with SYBR Green Master Mix (Roche Applied Science, Basel, Switzerland). The qPCR was performed on the ABI Prism 7500 Real-Time PCR System (Applied Biosystems, Foster City, CA, United States). PCR reaction conditions were as follows: 95°C for 30 s, then 55 cycles of 95°C for 10 s, 60 C for 30 s. The 2^−ΔΔCq^ method was used for comparative quantitation. The expression levels of target genes were determined relative to glyceraldehyde-3-phosphate dehydrogenase (*GAPDH*/*Gapdh*) using primers listed in Supplementary Table S1, which were designed using Primer Premier 5.0 software. Specifically, expression levels of human genes [collagen type I alpha 1 (*COL1A1*)*,* runt-related transcription factor 2 (*RUNX2*)*,* and Sp7 Transcription Factor (*SP7/OSX*)] and mouse genes (acid phosphatase 5, tartrate resistant (*Acp5*)*,* matrix metalloproteinase 9 (*Mmp9*)*,* cathepain K (*Ctsk*) and nuclear factor 1 of activated T cells (*Nfatc1*)] were assessed. All PCR reactions were performed in triplicate.

### 2.9 Western blot analysis

We utilized radioimmunoprecipitation assay lysis buffer (Solarbio), which was supplemented with a protease inhibitor mixture (Solarbio), to extract total protein from the cultured cells. Total protein (25 μg) was separated using 10% SDS–polyacrylamide gel, and proteins were transferred to polyvinyli-dene difluoride (PVDF) membranes (Millipore, Massachusetts, United States) (Xu et al., 2022). Then, we employed a solution of 0.1% Tween-20% and 5% non-fat milk to obstruct the membranes for a duration of 1 h. The membranes were incubated overnight at 4°C with the primary antibodies according to previous studies as follows (Ye et al., 2021; Chen et al., 2022b; He et al., 2022; Shen et al., 2023): anti-Piezo1 (1:1,000, 15939-1-AP; Proteintech, Cook, Illinois, United States), anti-COL1A1 (1:1,000, ab260043; Abcam), anti-Runx2 (1:1,000, ab236639; Abcam), anti-Osx (1:1,000, ab209484; Abcam), anti-GAPDH (1:3,000, 60004-1-Ig, ProteinTech), and anti-β actin antibodies (1:3,000, 81115-1-RR, ProteinTech). Then, the membranes were subjected to incubation with a horseradish peroxidase-conjugated mouse or rabbit IgG (1:5,000; Zhongshan Golden Bridge Biotechnology, Beijing, China). Super Signal West Pico Chemiluminescent Substrate (Thermo) and BioMax film (Kodak, Rochester, New York, United States) were used to detect the immunoreactive proteins. We quantified the relative density of three comparable results by utilizing the ImageJ software (National Institutes of Health, Bethesda, MD, United States). The analysis of immunoreactive bands employed GAPDH or β-actin as the control reference.

### 2.10 Immunofluorescence

hPDLCs seeded on chamber slides were subjected to force and/or LIPUS, followed by osteogenic induction for 7 days. The cells from different groups underwent fixation at 4°C for 15 min using 4% polyformaldehyde (PFA, Solarbio), followed by permeabilization for 5 min using 0.1% Triton X-100 (Solarbio). After a PBS wash, the cells were subjected to blocking with 5% bovine serum albumin (BSA, Solarbio) at room temperature for 60 min. Subsequently, the cells were subjected to immunostaining, employing a primary antibody, anti-Piezo1 (1:200, 15939-1-AP; Proteintech), overnight at 4°C. Following this, the cells were incubated with a Cy3 fluorescent secondary antibody (1:200, SA00009-2, Proteintech) at room temperature for 1 h. Subsequently, after PBS washing, the cells were sealed using a mounting medium containing 4′,6-diamidino-2-phenylindole (DAPI, Solarbio). Images were captured using an inverted confocal microscope (FV3000, Olympus, Tokyo, Japan).

### 2.11 Rat OTM model

**FIGURE 1:**
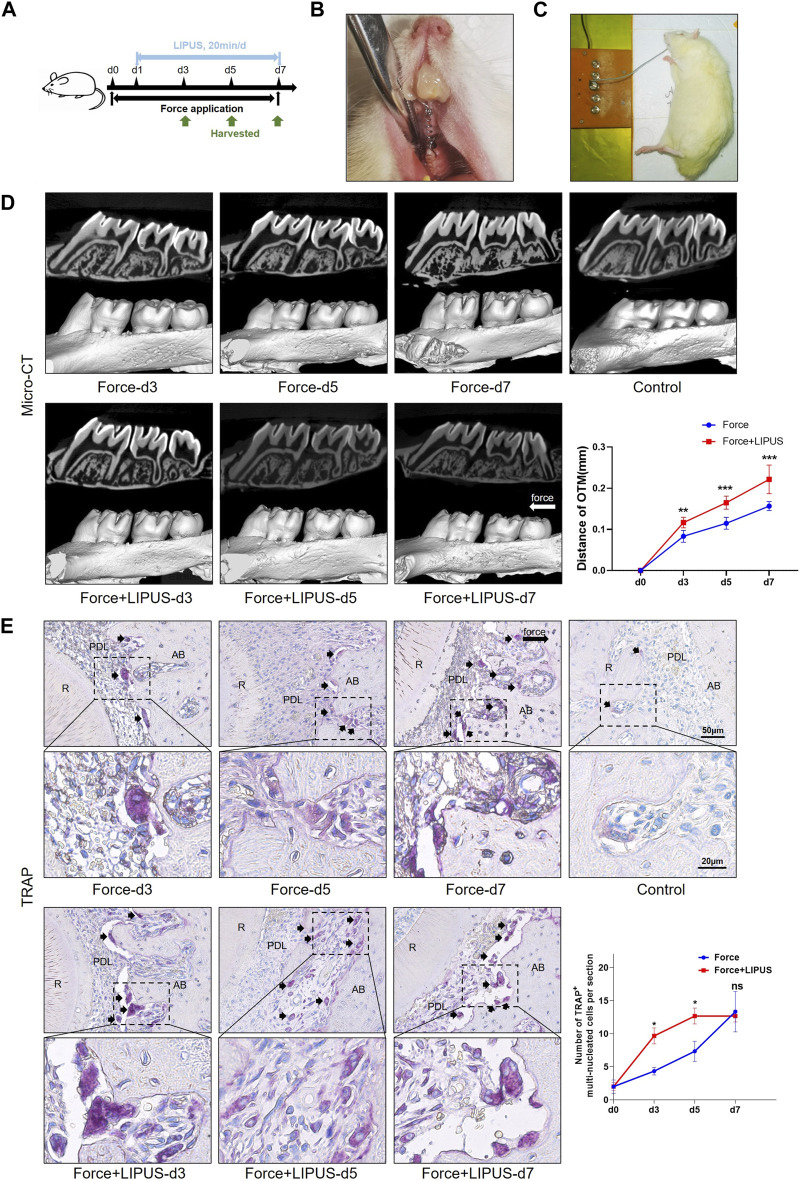
LIPUS promoted osteoclastic differentiation and accelerated OTM in rats **(A)** Schedule diagram of the experiment. Orthodontic force was applied to rats in the Force and Force + LIPUS groups for 3, 5 or 7 days. One day after establishing the OTM model (d1), rats in the Force + LIPUS group were subjected to daily LIPUS stimulation for 20 min. **(B)** Representative image of the experimental rats during the OTM procedure. **(C)** Representative image of daily LIPUS stimulation. **(D)** Representative micro-CT images of tooth movement and semi-quantitative analysis of OTM distance. The direction of the orthodontic force is indicated by the white arrow (*n* = 6). **(E)** Representative tartrate-resistant acid phosphatase (TRAP) staining images of the distobuccal root’s compression side. The long black arrow indicates the force direction. The short black arrows indicate TRAP-positive multinucleated osteoclasts. The semi-quantitative assessment demonstrates that the Force + LIPUS group exhibited a higher quantity of osteoclasts on the pressure side compared to the Force group on the third and fifth days. However, by the seventh day, there was no significant difference observed between the Force and Force + LIPUS groups. Scale bar: 50 μm (up) and 20 μm (down) (*n* = 3). Data are represented as mean with 95% confidence interval. Student’s t-test was performed: ***p < 0.001; *p < 0.05; and ns, not significant (*p* > 0.05). LIPUS, low-intensity pulsed ultrasound; OTM, orthodontic tooth movement; R, the distal buccal root of the maxillary first molar; PDL, periodontal ligament; AB, alveolar bone.

Six-week-old male Sprague-Dawley (SD) rats weighing 200 ± 20 g were purchased from Beijing Vital River Laboratory Animal Technology Co., Ltd. (Beijing, China). The rats were anesthetized using pentobarbital sodium (40 mg/kg of body weight) for the application of orthodontic devices. A rat OTM model was established as described before (Xin et al., 2023), with a closed-coil spring (Tomy International, Inc. ,Tokyo, Japan) ligated to the right maxillary first molar and incisor neck via a 0.025 mm diameter stainless-steel ligation wire (Tomy International, Inc.). The spring applied a force of 50 g to move the upper first molar mesially, and we ensured that the force applied to each rat was consistent using a force gauge (3M Unitek, United States) (Jin et al., 2020; Jiang et al., 2021). The experimenters involved in constructing the OTM model were blinded to the group allocation, ensuring the precise application of force. To enhance the retaining force and prevent the device from falling off, a 0.5 mm deep groove was made using a slow-speed mill near the gingival margin of the maxillary incisor to accommodate the ligature wire and filled with flowing resin (3M Unitek) (Figure 1B). After all rats regained consciousness, they were fed soft food daily.

### 2.12 Grouping for experimental treatments

One hundred and eight SD rats were involved in this study. 60 SD rats were enrolled in the LIPUS function assay and were divided into 10 groups (Control; LIPUS, Force and Force + LIPUS at day 3, 5, and 7) using random drawing of lots, and each group consisting of 6 rats.

Twenty-four SD rats were enrolled in the Piezo1 functional inhibition assay and were evenly divided into 4 groups using random drawing of lots. Based on the established experimental method (Jiang et al., 2021), the Force + GsMTx4 and Force + GsMTx4 + LIPUS groups received a subcutaneous injection behind the neck of the Piezo1 inhibitor, Grammostola spatulata mechanotoxin 4 (GsMTx4, P1205, Selleck, Houston, TX, United States), at a dosage of 100 μL/kg with a concentration of 10 μM every other day, while the other groups received only the vehicle (PBS).

Twenty-four SD rats were enrolled in the Piezo1 functional activation assay and were evenly divided into 4 groups using random drawing of lots. The animals in the Yoda and Yoda + LIPUS groups received an intraperitoneal injection of Piezo1 agonist Yoda1 (S6678, Selleck, Houston, TX, United States) at a dosage of 100 μL/kg with a concentration of 25 μM (diluted in PBS) every other day according to the instruction of the manufacturer and the effective concentration for *in vitro* experiments, whereas the other groups received only the vehicle (DMSO + PBS) (Zeng et al., 2022; Zhang et al., 2023a).

LIPUS was applied after mechanical loading had been exerted on the right maxillary first molars for 20 min each day before harvesting. After general anesthesia, the skin overlying the first molar sites was placed on the ultrasound transducer with a thin layer of standard ultrasound gel (Figure 1C). The sham controls underwent the same handling procedures as the treated rats, but the ultrasound generator was not turned on. After the experiment was completed, the experimenters waited for all animals to fully regain consciousness and restore their normal activity before concluding the experiment and returning them to their appropriate housing environments.

### 2.13 Micro-computed tomography (micro-CT) imaging and analysis

After 7 days of treatment, the rats (*n* ≥ 3) were sacrificed via overdose of pentobarbital sodium, and the maxillae were harvested and fixed in 4% PFA. Subsequently, we acquired micro-CT images of the maxillae at an 8.82 μm resolution, utilizing a tube voltage of 80 kV, a tube current of 500 μA, and an exposure time of 1,500 ms. Three-dimensional (3D) reconstructions were performed using a multimodal 3D visualization software (Inveon Research Workplace; Siemens, Munich, Germany) (Xin et al., 2023). The measurement of tooth movement resulting from mechanical loading adopted a modified technique outlined in an earlier work (Xin et al., 2023). In brief, two proficient investigators, both unaware of the group allocation, measured the distance between two easily identifiable points: the midpoint of the distal marginal ridge of the first molar and the midpoint of the mesial marginal ridge of the second molar. The mean of these dual measurements was used to calculate the distance of tooth movement.

To assess changes in alveolar bone density on the pressure side, a square-shaped region of interest (ROI) measuring 0.3 mm × 1 mm × 1 mm in volume was selected at the mesial aspect of the distobuccal root of the maxillary first molar after three-dimensional reconstruction (Supplementary Figure S3). The bone volume fraction (BV/TV) and trabecular morphology parameters, including trabecular number (Tb.N), trabecular thickness (Tb.Th), and trabecular separation (Tb.Sp), were analyzed within this ROI. Each sample was analyzed three times by two proficient investigators, who were blinded to the group allocation. The mean of these measurements was considered the final measurement for each sample.

### 2.14 Hematoxylin and eosin and tartrate-resistant acid phosphatase staining

After fixation, the maxillae underwent demineralization in 15% ethylenediaminetetraacetic acid and were then embedded in paraffin blocks. Subsequently, 4 μm thick slices perpendicular to the root axis were obtained from their respective experimental groups; the slides were subjected to the staining procedures involving both hematoxylin and eosin (H&E) as well as tartrate-resistant acid phosphatase (TRAP). The observed area was the periodontal ligament (PDL) on the pressure side of the distal buccal root of the maxillary first molar. For TRAP staining, an acid phosphatase kit (387A-1KT; Sigma) was employed, and the guidelines provided by the manufacturer were strictly followed. We conducted quantification of TRAP-positive multi-nucleated cells (≥3 nuclei) along the entire surface of the alveolar bone’s pressure side of each slice.

### 2.15 Immunohistochemistry

The slices were subjected to antigen retrieval using 0.125% trypsin (Gibco) and 20 μg/mL of proteinase K solution (Invitrogen, Carlsbad, CA, United States) at 37°C for 1 h. The slides were blocked with 5% BSA for 30 min at room temperature, followed by overnight incubation at 4°C with the primary antibodies, including anti-Piezo1 (1:100, 15939-1-AP; Proteintech), anti-COL1A1 (1:200, ab260043; Abcam), anti-osteocalcin (anti-OCN; 1:100, 23418-1-AP; Proteintech) antibodies. After thorough PBS washing, the slides were exposed to HRP-conjugated secondary antibodies and visualized using diaminobenzidine (Zhongshan Golden Bridge Biotechnology) as per the manufacturer’s instructions. Subsequently, the slides were counterstained with H&E and examined using a light microscope (BX53, Olympus, Tokyo, Japan). The Image-Pro Plus 6.0 Software (Media Cybernetics, Bethesda, MD, United States) was employed for the analysis of positive cells after IHC staining.

### 2.16 Statistical analysis

The analysis of statistical data was executed using the Statistical Package for the Social Sciences 19.0 software. The data were presented as mean values with 95% confidence interval. The Shapiro-Wilk test was employed to verify the normal distribution of the original dataset, while the evaluation of significance relied on both two-tailed independent Student’s t-test and one-way analysis of variance (ANOVA). To further compare the ANOVA results, Tukey’s multiple comparison test was adopted. Statistical significance was attributed to differences where *p*-value was less than 0.05.

## 3 Results

### 3.1 LIPUS promoted osteoclastic differentiation and accelerated OTM in rats

Following the establishment of the OTM model, the rats in the Force + LIPUS group received daily LIPUS stimulation for 20 min (Figures 1A–C). LIPUS accelerated OTM, evident from the third day of treatment (Figure 1D). The H&E staining results indicated that compared to the control group, the fiber arrangement was disordered in the Force group and the Force + LIPUS group (Supplementary Figure S2). TRAP staining results indicated an increase in the number of TRAP-positive osteoclasts accumulated in the periodontal tissues on the compression side during both mechanical loading and LIPUS application. On the Day 3 and Day 5, the Force + LIPUS group exhibited a higher quantity of osteoclasts on the pressure side compared to the Force group. However, by the seventh day, there was no significant difference observed between the Force and Force + LIPUS groups (Figure 1E).

### 3.2 LIPUS reduced force-induced alveolar bone resorption in rats

**FIGURE 2:**
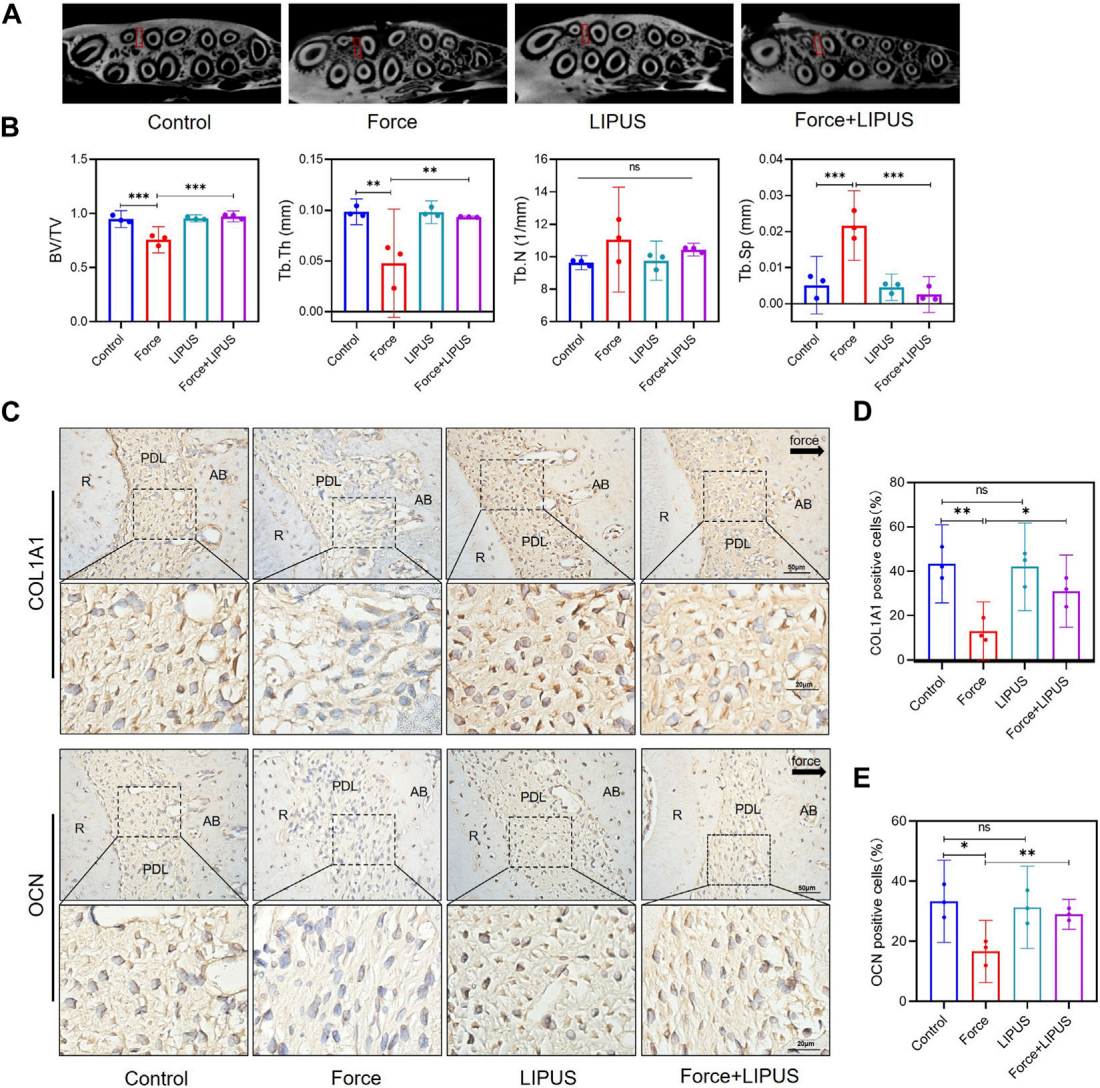
LIPUS reduced force-induced alveolar bone resorption in rats **(A)** Representative micro-CT images of with delineated region of interest (ROI, marked with red rectangular boxs) of cross-sections. **(B)** semi-quantitative evaluation of bone volume fraction (BV/TV), trabecular number (Tb.N), trabecular thickness (Tb.Th) and trabecular separation (Tb.Sp) (*n* = 6). **(C–E)** Representative immunohistochemical staining images and semi-quantitative evaluation of COL1A1-and OCN-positive cells in the periodontal tissues post-force and post-LIPUS application on the seventh day. The direction of mechanical force is indicated by the black arrow. Scale bar: 50 μm (up) and 20 μm (down) (*n* = 3). Data are represented as mean with 95% confidence interval. One-way ANOVA with Tukey’s test was performed: ***p < 0.001; **p < 0.01; *p < 0.05; and ns, not significant (*p* > 0.05). LIPUS, low-intensity pulsed ultrasound; R, the distal buccal root of the maxillary first molar; PDL, periodontal ligament; AB, alveolar bone.

Given the absence of a substantial variance in the number of osteoclasts differentiated on the pressure side between the Force group and the Force + LIPUS group on the seventh day, we seek to delve deeper into the potential influence of LIPUS on the bone deposition. Micro-CT analysis indicated that LIPUS reduced alveolar bone resorption after force application (Figures 2A, B). LIPUS enhanced the expression of COL1A1 and OCN in the PDL on the pressure side. But there was no significant difference on the number of COL1A1-positive cells and OCN-positive cells between Control group and LIPUS group (Figures 2C–E).

### 3.3 LIPUS promoted osteogenic differentiation of force-treated hPDLCs

According to the results of the flow cytometry analysis, the surface markers CD31, CD45 and CD34 were negative, while CD29, CD90 and CD105 were positive (Supplementary Figure S4).

**FIGURE 3:**
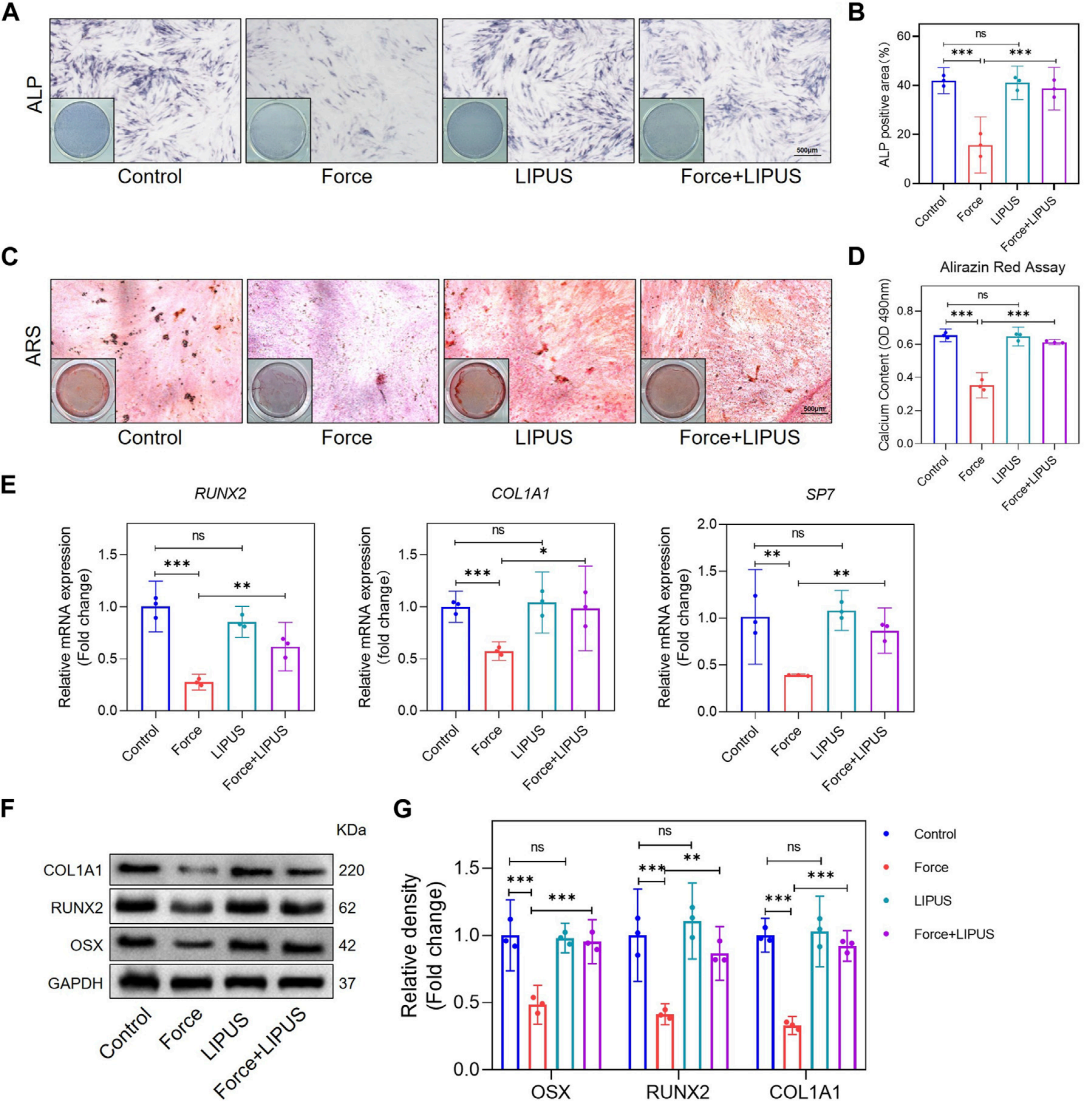
LIPUS promoted osteogenesis of force-treated hPDLCs **(A,B)** Representative images and semi-quantitative analysis of ALP staining of hPDLCs after osteogenic induction for 7 days. Scale bar = 500 μm (*n* = 3). **(C,D)** Representative images of Alizarin Red staining and CPC quantification of hPDLCs after osteogenic induction for 21 days. Scale bar = 500 μm (*n* = 3). **(E)** Real-time quantitative PCR analysis of the mRNA expression levels of *RUNX2, COL1A1* and *SP7*. **(F,G)** Representative images of Western blot and semi-quantitative analysis of the protein levels of RUNX2, COL1A1 and OSX. Data are represented as mean with 95% confidence interval. One-way ANOVA with Tukey’s test was performed: ***p < 0.001; **p < 0.01; *p < 0.05; and ns, not significant (*p* > 0.05). All experiments were repeated three times. LIPUS, low-intensity pulsed ultrasound; hPDLCs, human periodontal ligament cells.

To elucidate the impact of LIPUS on the osteogenic differentiation of force-treated hPDLCs, we conducted ALP staining and Alizarin Red staining at 7 and 21 days after osteogenic induction, respectively. Compared to the control group, mechanical force hindered the osteogenic differentiation of hPDLCs. However, the Force + LIPUS group exhibited a higher level of ALP activity (Figures 3A, B) and an increased number of mineralized nodules compared to the Force group (Figures 3C, D). In the Force + LIPUS group, the mRNA levels of osteogenic markers, including *SP7, RUNX2* and *COL1A1*, were significantly higher than those in the Force group (Figure 3E). The Western blot analysis further revealed a significant increase in the protein expression levels of OSX, RUNX2 and COL1A1 in force-treated hPDLCs following LIPUS stimulation (Figures 3F, G). These findings confirmed that LIPUS promoted the osteogenesis of force-treated hPDLCs.

To investigate the influence of LIPUS on *in vitro* osteoclast differentiation, supernatants from hPDLCs subjected to distinct treatments were collected. Subsequently, conditioned medium was prepared to observe its effects on osteoclast differentiation of RAW264.7 cells. Semi-quantitative analysis indicates that both Force and Force + LIPUS treatments enhance osteoclast differentiation. However, there is no significant difference in the number of osteoclasts between the CM-Force group and CM-Force + LIPUS group (Supplementary Figures S5A, B). The mRNA expression patterns of *Acp5, Mmp9, Ctsk* and *Nfatc1* align with the findings from TRAP staining (Supplementary Figure S5C).

### 3.4 LIPUS downregulated Piezo1 in force-treated PDLCs *in vitro* and *in vivo*

**FIGURE 4:**
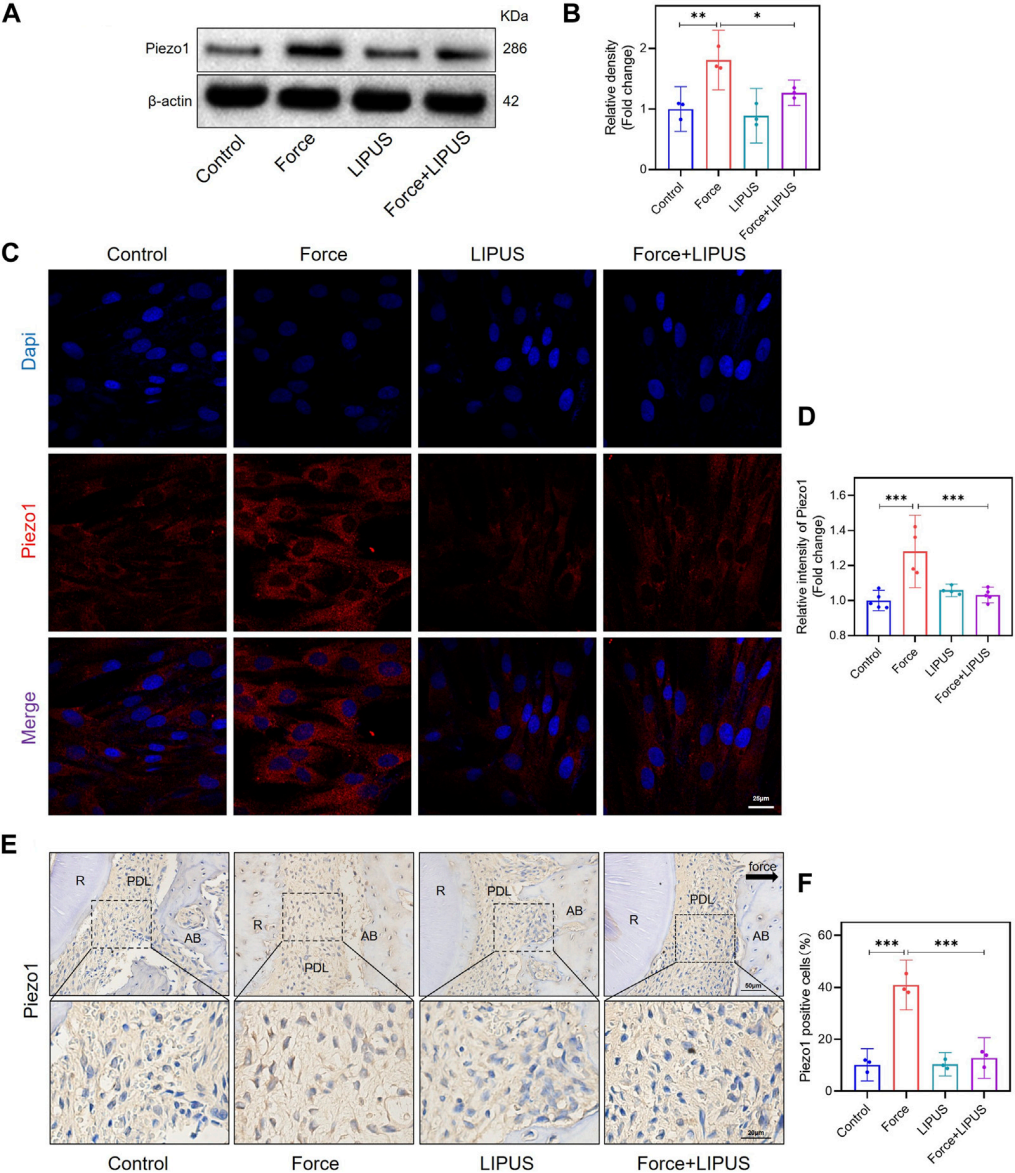
LIPUS downregulated Piezo1 in force-treated PDLCs *in vitro* and *in vivo*. **(A,B)** Representative images of Western blot and semi-quantitative analysis of Piezo1 protein level in hPDLCs after osteogenic induction for 7 days. **(C,D)** Representative images and semi-quantitative analysis of the average fluorescence intensity of Piezo1 immunofluorescence staining in hPDLCs after osteogenic induction for 7 days. Scale bar: 25 μm (*n* ≥ 4). **(E,F)** Representative immunohistochemical staining images and semi-quantitative evaluation of Piezo1 in the periodontal tissues post-force and post-LIPUS application. The direction of mechanical force is indicated by the black arrow. Scale bar: 50 μm (up) and 20 μm (down) (*n* = 3). Data are represented as mean with 95% confidence interval. One-way ANOVA with Tukey’s test was conducted: ***p < 0.001; **p < 0.01; and *p < 0.05. All experiments were repeated three times. LIPUS, low-intensity pulsed ultrasound; hPDLCs, human periodontal ligament cells; R, the distal buccal root of the maxillary first molar; PDL, periodontal ligament; AB, alveolar bone.

Since Piezo1 can respond to mechanical or ultrasonic stimulation (Zhang et al., 2023b; Du and Yang, 2023; Schröder et al., 2023), we identified changes in Piezo1 expression in hPDLCs after force and LIPUS treatment *in vitro* and in the pressure side PDL of rats. The Western blot analysis revealed a significant increase in Piezo1 expression in force-treated hPDLCs, but LIPUS downregulated the Piezo1 (Figures 4A, B). The results of the immunofluorescence staining analysis were consistent with the Western blot analysis (Figures 4C, D). The immunohistochemical staining of Piezo1 in the periodontal tissues following force and LIPUS application revealed that LIPUS downregulated the number of Piezo1-positive cells in the force-treated periodontal tissues (Figures 4E, F). By comparing the results of the control group and the LIPUS group, we found that LIPUS stimulation alone did not alter piezo1 expression, which was consistent with the changes in osteogenic phenotype.

### 3.5 Knockdown of Piezo1 promoted the osteogenesis of force-treated hPDLCs and partially blocked the effect of LIPUS

**FIGURE 5:**
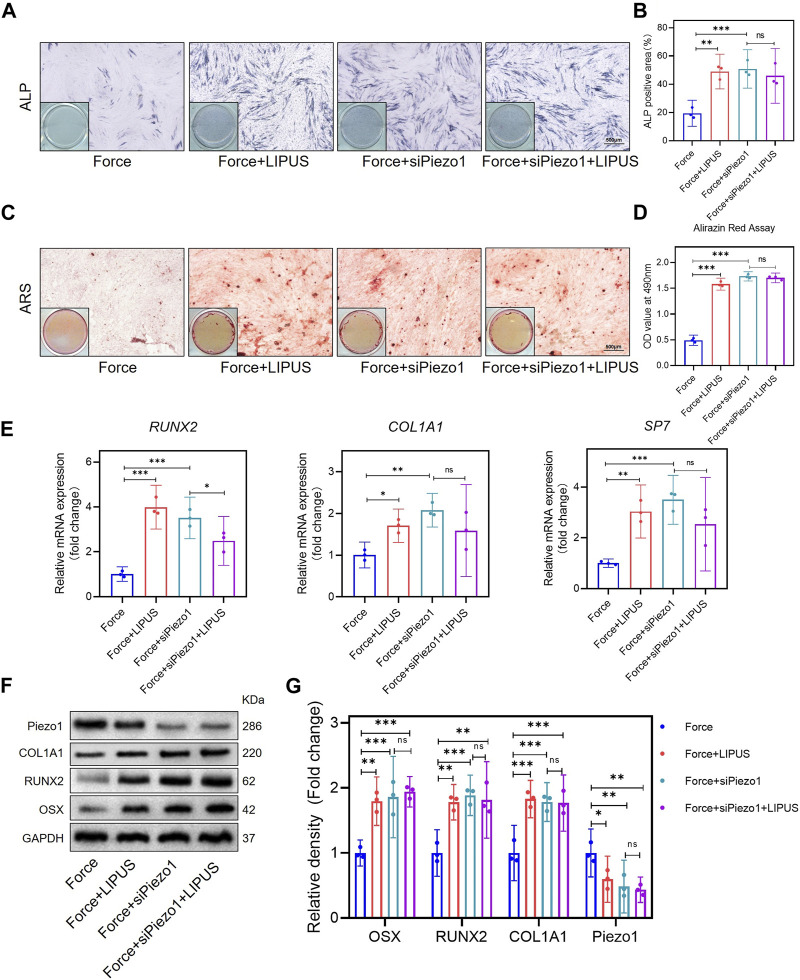
Knockdown of Piezo1 promoted the osteogenesis of force-treated hPDLCs and blocked the effect of LIPUS **(A,B)** Representative images and semi-quantitative analysis of ALP staining of hPDLCs after osteogenic induction for 7 days. Scale bar = 500 μm (*n* = 3). **(C,D)** Representative images of Alizarin Red staining and CPC quantification of hPDLCs after osteogenic induction for 21 days. Scale bar = 500 μm (*n* = 3). **(E)** Real-time quantitative PCR analysis of the mRNA expression levels of *RUNX2, COL1A1,* and *SP7*. **(F,G)** Representative images of Western blot results show that changes in and semi-quantitative analysis of the protein levels of RUNX2, COL1A1, OSX and Piezo1. Data are represented as mean with 95% confidence interval. One-way ANOVA with Tukey’s test was performed: ***p < 0.001; **p < 0.01; *p < 0.05; and ns, not significant (*p* > 0.05). All experiments were repeated three times. LIPUS, low-intensity pulsed ultrasound; hPDLCs, human periodontal ligament cells.

After the knockdown of Piezo1 in hPDLCs, osteogenic differentiation was promoted in force-treated hPDLCs. The Force + siPiezo1 group exhibited a higher level of ALP activity (Figures 5A, B) and an increased number of mineralized nodules compared to the Force group (Figures 5C, D). The mRNA levels of the osteogenic markers *SP7, RUNX2* and *COL1A1* were significantly higher in the Force + siPiezo1 group than in the Force group (Figure 5E). The Western blot analysis also showed that the protein expression levels of OSX, RUNX2 and COL1A1 were significantly increased in the Force + siPiezo1 group after the reduction in Piezo1 in force-treated hPDLCs (Figures 5F, G). No significant difference was observed in the levels of mRNA and protein related to osteogenesis between the Force + siPiezo1 group and the Force + siPiezo1+LIPUS group (Figures 5E–G). These results suggested that the reduction in piezo1 partially attenuated the effects of LIPUS.

### 3.6 GsMTx4 inhibited OTM, alleviated force-induced bone resorption and partially blocked the effects of LIPUS

To explore whether Piezo1 plays an active role in the process of OTM and the effectiveness of LIPUS, we conducted a functional inactivation trial (Supplementary Figure S7). GsMTx4 has been reported in previous research to be capable of inhibiting mechanical-sensitive cation channels (Bae et al., 2011).

**FIGURE 6:**
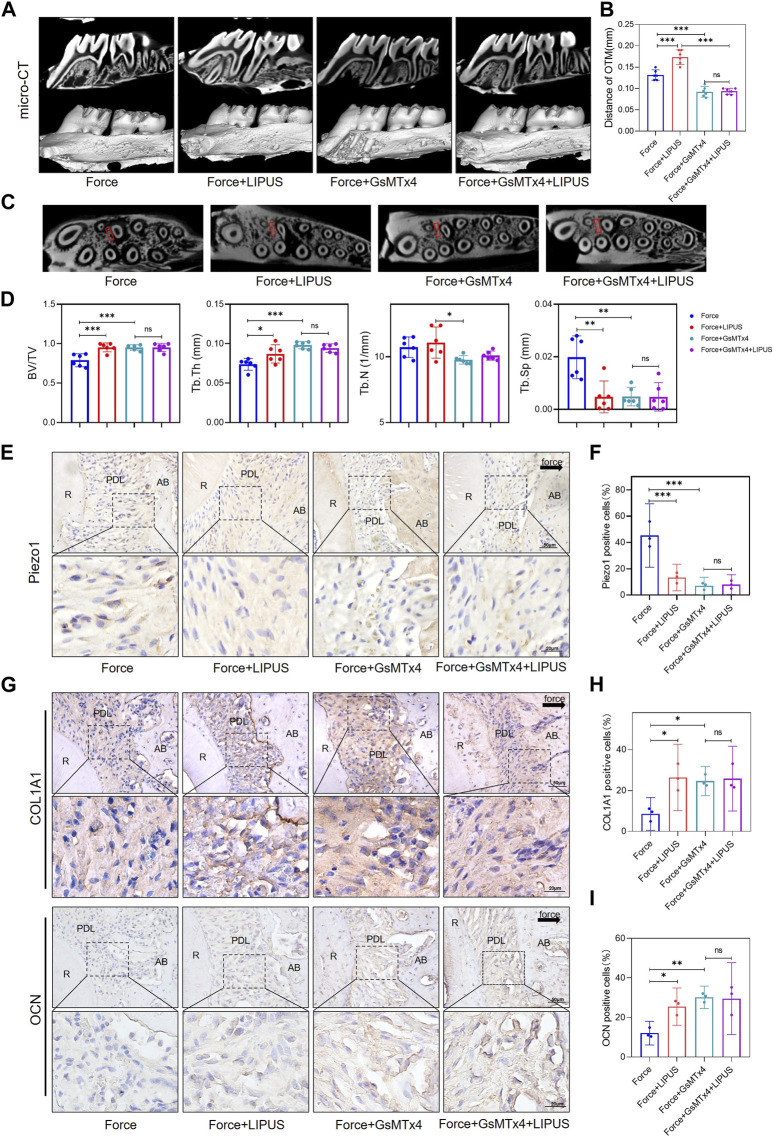
GsMTx4 inhibited OTM, alleviated force-induced bone resorption and partially blocked the effects of LIPUS **(A,B)** Representative micro-CT images of tooth movement and semi-quantitative analysis of OTM distance. The direction of the orthodontic force is indicated by the white arrow (*n* = 6). **(C)** Representative micro-CT images with delineated region of interest (ROI, marked with red rectangular boxs) of cross-sections. **(D)** semi-quantitative evaluation of bone volume fraction (BV/TV), trabecular number (Tb.N), trabecular thickness (Tb.Th) and trabecular separation (Tb.Sp) (*n* = 6). **(E,F)** Representative immunohistochemical staining images and semi-quantitative evaluation of Piezo1 expression in the periodontal tissues. The direction of mechanical force is indicated by the black arrow. Scale bar: 50 μm (up) and 20 μm (down) (*n* = 3). **(G–I)** Representative immunohistochemical staining images and semi-quantitative evaluation of COL1A1-positive and OCN-positive cells in the periodontal tissues. The direction of mechanical force is indicated by the black arrow. Scale bar: 50 μm (up) and 20 μm (down) (*n* = 3). Data are represented as mean with 95% confidence interval. One-way ANOVA with Tukey’s test was conducted: ***p < 0.001; **p < 0.01; *p < 0.05; and ns, not significant (*p* > 0.05). LIPUS, low-intensity pulsed ultrasound; OTM, orthodontic tooth movement; R, the distal buccal root of the maxillary first molar; PDL, periodontal ligament; AB, alveolar bone.

The micro-CT analysis indicated that GsMTx4 effectively inhibited OTM and alleviated bone resorption induced by orthodontic force. And the effect of LIPUS on accelerating OTM was also blocked (Figures 6A–D). Immunohistochemistry staining confirmed that Piezo1 was inhibited by the injection of GsMTx4 (Figures 6E, F). An increased number of COL1A1-and OCN-positive cells was observed in the PDL on the compression side of both the Force + GsMTx4 group and the Force + GsMTx4+LIPUS group compared to the Force group, but there was no significant difference between the Force + GsMTx4 group and the Force + GsMTx4+LIPUS group (Figures 6G–I).

### 3.7 LIPUS promoted the osteogenesis of Yoda1-treated hPDLCs

**FIGURE 7:**
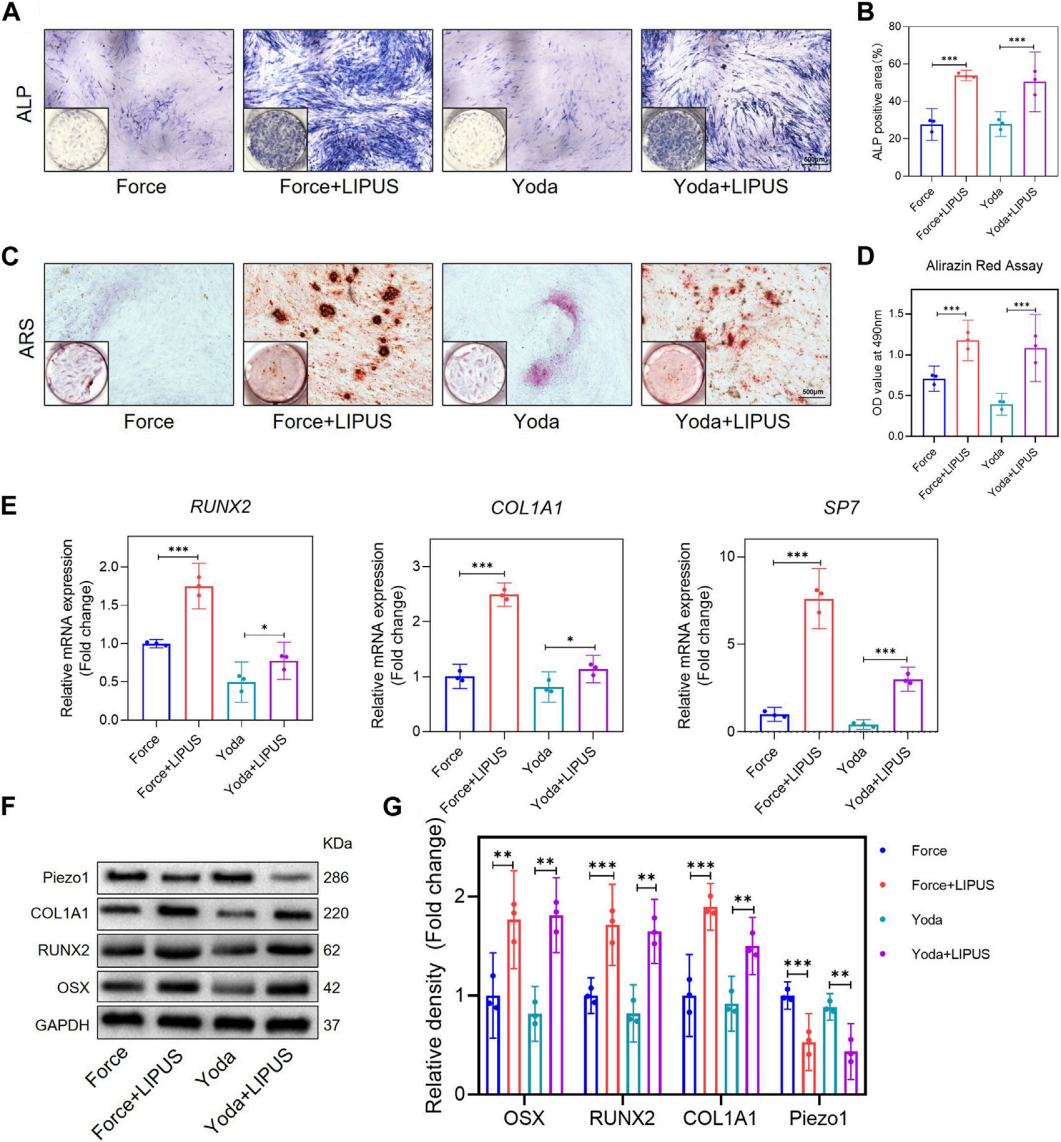
LIPUS promoted the osteogenesis of Yoda1-treated hPDLCs **(A,B)** Representative images and semi-quantitative analysis of ALP staining of hPDLCs after osteogenic induction for 7 days. Scale bar = 500 μm (*n* = 3). **(C,D)** Representative images of Alizarin Red staining and CPC quantification of hPDLCs after osteogenic induction for 21 days. Scale bar = 500 μm (*n* = 3). **(E)** Real-time quantitative PCR analysis of the mRNA expression levels of *RUNX2, COL1A1* and *SP7*. **(F,G)** Representative images of Western blot results show that changes in and semi-quantitative analysis of the protein levels of RUNX2, COL1A1, OSX and Piezo1. Data are represented as mean with 95% confidence interval. One-way ANOVA with Tukey’s test was conducted: ***p < 0.001 and **p < 0.01. All experiments were repeated three times. LIPUS, low-intensity pulsed ultrasound; hPDLCs, human periodontal ligament cells.

Yoda1 has been reported to be capable of activating purified Piezo1 channel (Botello-Smith et al., 2019). To further explore the role of Piezo1 in the LIPUS application process, hPDLCs were pre-stimulated with Yoda1 before osteogenic induction. The osteogenic differentiation of Yoda1-treated hPDLCs was enhanced following LIPUS treatment, as evidenced by increased ALP activity (Figures 7A, B) and augmented mineralization nodules (Figures 7C, D). The alterations in the mRNA levels of *SP7, RUNX2,* and *COL1A1* in the Yoda group and the Yoda + LIPUS group also proved this (Figure 7E). The protein level of Piezo1 in the Yoda group was upregulated, while it was downregulated in the Yoda + LIPUS group. Simultaneously, the western blot analyses demonstrated a significant decrease in the expression levels of OSX, RUNX2 and COL1A1in the Yoda group, with the LIPUS intervention reversing this effect (Figures 7F, G). These changes closely resembled those observed in hPDLCs after mechanical force treatment and LIPUS stimulation.

### 3.8 LIPUS promoted the osteogenesis of PDLSCs in Yoda1-treated rats

**FIGURE 8:**
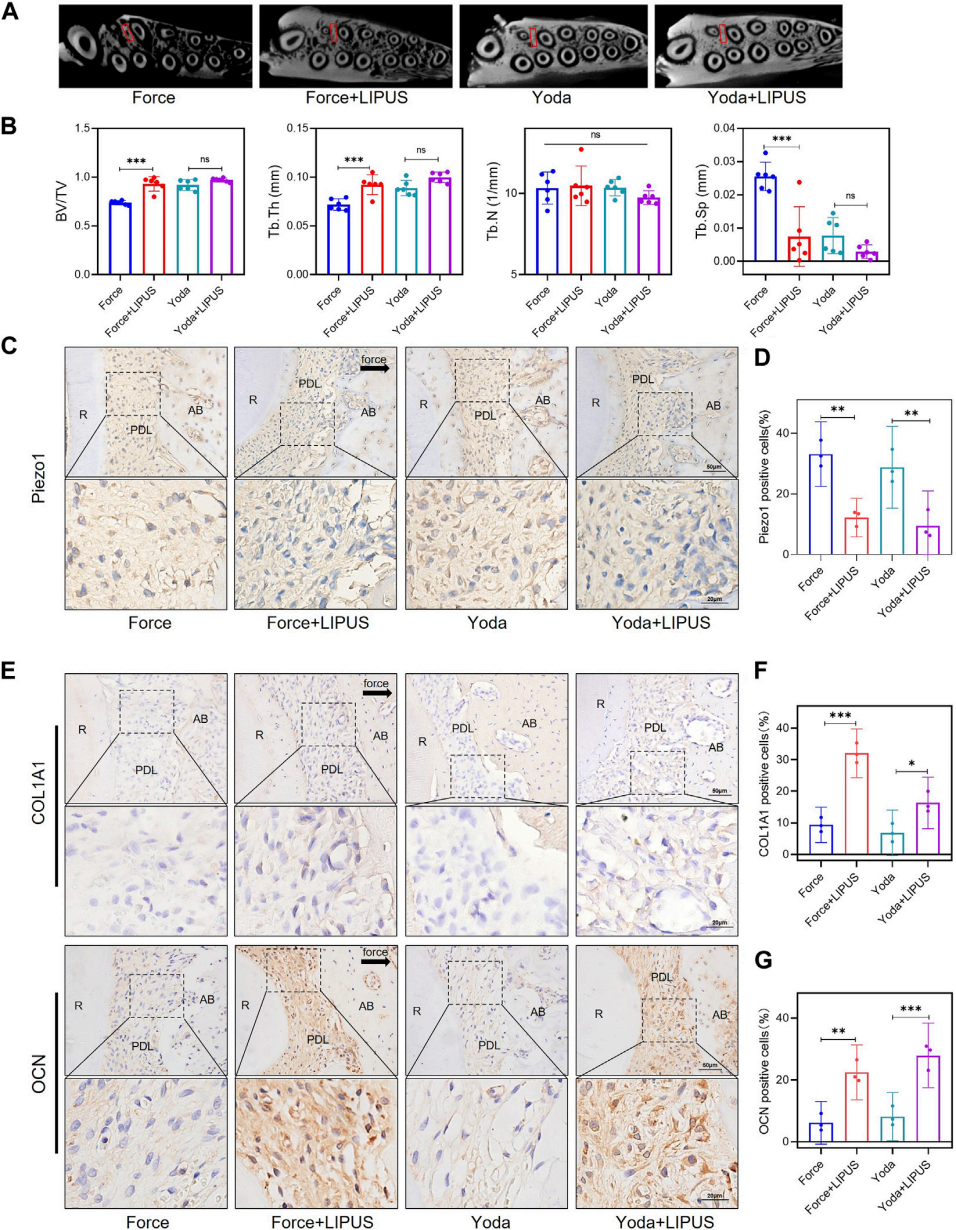
LIPUS promoted the osteogenesis of PDLCs in Yoda1-treated rats **(A)** Representative micro-CT images of with delineated region of interest (ROI, marked with red rectangular boxs) of cross-sections. **(B)** semi-quantitative evaluation of bone volume fraction (BV/TV), trabecular number (Tb.N), trabecular thickness (Tb.Th) and trabecular separation (Tb.Sp) (*n* = 6). **(C,D)** Representative immunohistochemical staining images and semi-quantitative evaluation of Piezo1 in the periodontal tissues. The direction of mechanical force is indicated by the arrow. Scale bar: 50 μm (up) and 20 μm (down) (*n* = 3). **(E–G)** Representative immunohistochemical staining images and semi-quantitative evaluation of COL1A1-and OCN-positive cells in the periodontal tissues. The direction of mechanical force is indicated by the black arrow, and the small boxes within the larger ones offer higher magnification views. Scale bar: 50 μm (up) and 20 μm (down) (*n* = 3). Data are represented as mean with 95% confidence interval. One-way ANOVA with Tukey’s test was conducted: ***p < 0.001; **p < 0.01; and *p < 0.05. LIPUS, low-intensity pulsed ultrasound; R, the distal buccal root of the maxillary first molar; PDL, periodontal ligament; AB, alveolar bone.

Yoda1 was administered to SD rats to investigate the role of Piezo1 in the context of LIPUS (Supplementary Figure S8). Micro-CT revealed that Yoda1 injections for 1 week did not affect alveolar bone mineral density. There was no difference in bone density between the Yoda and Yoda + LIPUS group (Figures 8A, B). The immunohistochemical staining confirmed the activation of Piezo1 following Yoda1 injection. Furthermore, LIPUS stimulation downregulated Piezo1 expression (Figures 8C, D). The Yoda + LIPUS group exhibited a higher number of COL1A1-and OCN-positive cells in the PDL on the “compression side” compared to the Yoda group (Figures 8E–G). These observed changes closely resembled those seen in PDLCs after the force treatment and LIPUS stimulation *in vivo*.

## 4 Discussion

In this study, we tested a novel mechanism that LIPUS promotes the osteogenic differentiation of force-treated PDLCs via downregulating Piezo1. Firstly, LIPUS can promote osteoclast differentiation on the pressure side in the early stages (day 3 and day 5) and accelerate OTM. Secondly, we observed that LIPUS effectively enhanced osteogenesis of force-treated PDLCs and reduced the alveolar bone resorption under pressure. Thirdly, it was noted that mechanical force activated Piezo1 expression in PDLCs, whereas LIPUS treatment led to a downregulation of Piezo1 expression. The inhibition of Piezo1 not only promoted the osteogenic differentiation of force-treated PDLCs but also attenuated the positive effects of LIPUS. Finally, we utilized Yoda1 to mimic the mechanical force-induced activation of Piezo1 in PDLCs. Notably, Yoda1 demonstrated an inhibitory effect on osteogenesis, while LIPUS was capable of mitigating this inhibition. Furthermore, it is interesting to note that LIPUS stimulation alone did not affect the osteogenic differentiation of PDLCs in our study.

The compressive side of OTM is notably marked by heightened osteoclastic activity (Wang et al., 2018; Jin et al., 2020). This prompts our interest in the potential of LIPUS as a therapeutic intervention to regulate this equilibrium and impact both osteogenic and osteoclastic activities. Our hypothesis is that LIPUS might play a role in regulating this dynamic equilibrium, which is crucial for maintaining the homeostasis of periodontal support tissues. A retrospective clinical study showed that LIPUS reduced the duration of invisible orthodontic treatment by an average of 49% (Kaur and El-Bialy, 2020). El-Bialy et al. reported the findings of a prospective multi-center randomized controlled trial, which showed that LIPUS not only shortened the orthodontic treatment duration but also minimized orthodontically induced tooth root resorption (El-Bialy et al., 2020). Additionally, several animal experiments showed that LIPUS could accelerate tooth movement in rats (Trackman et al., 2013; Alazzawi et al., 2018; Arai et al., 2020). The effect of LIPUS on accelerating tooth movement may be attributed to its promotion of osteoclast differentiation (Trackman et al., 2013; Feres et al., 2016; Zhou et al., 2023). In this study, LIPUS enhances osteoclastic differentiation and accelerates OTM from the third day, which is consistent with other research (Zhou et al., 2023). However, there is no significant difference in the number of TRAP-positive osteoclasts accumulating at the alveolar bone between the Force group and the Force + LIPUS group on day 7.

PDLCs play crucial roles in maintaining periodontal homeostasis and regulating periodontal tissue remodeling. They possess diverse local functions, such as the formation and preservation of periodontal ligaments, facilitation of alveolar bone and cementum repair, and contribution to periodontal tissue regeneration (Seo et al., 2004; Jiang et al., 2021). In this study, hPDLCs were isolated following methods outlined in previous literature (Jin et al., 2020; Xie et al., 2022), sourced from three donors of varying ages (Host 1: 14-year-old, male; Host 2: 17-year-old, female; Host 3: 21-year-old, female). This approach was implemented to mitigate, to some extent, the potential influence of age-related factors on phenotypic outcomes. The process of mechanical force-induced periodontium remodeling and reconstruction necessitates a harmonious interplay between osteoclast-mediated bone resorption and osteoblast-mediated bone formation (Wang et al., 2018). In our study, we observed a reduction in the osteogenic differentiation ability of force-treated PDLCs. These findings align with previous research conducted by other scholars. Jin et al. conducted research involving rat PDLSCs subjected to mechanical force *in vivo*, revealing a lower level of formation of mineralization crystals during *in vitro* osteogenesis induction in force-treated rat PDLSCs when compared to those not subjected to the force treatment (Jin et al., 2020). Furthermore, *in vitro* experiments involving the application of static compressive stimuli for 24 h resulted in a reduction in the expression of collagen Ⅰ in human PDLSCs (Feng et al., 2016). Notably, one-hour hydraulic pressure stimulation promoted the osteogenesis of PDLSCs, whereas 12-h hydraulic pressure stimulation inhibited their osteogenic potential (Zhang et al., 2016). Cyclic stress enhanced the osteogenic differentiation of PDLSCs (Zhang et al., 2023c). These collective findings suggest that short-term static pressure stimulation and periodic pressure stimulation have the potential to enhance the osteogenic differentiation of PDLSCs, whereas prolonged static pressure stimulation may hinder this process. In this study, static pressure was only applied to hPDLCs *in vitro* for 24 h, which did not fully simulate the sustained stress state of the pressure side PDL in the rat OTM model. In the future, we aim to explore a more compatible method of force application.

LIPUS promotes the osteogenesis of various cells, such as BMSCs (Liang et al., 2022; Yao et al., 2022), periosteal cells (Maung et al., 2021), and PDLSCs (Li et al., 2020a). In a previous study, LIPUS was found to enhance the osteogenesis of hPDLSCs from periodontitis donors (Li et al., 2020a). Our study also revealed that LIPUS enhances the osteogenic potential of force-treated PDLCs, which may provide the insights into the mechanism underlying its ability to facilitate alveolar bone regeneration and mitigate tooth root resorption during orthodontic treatment. Our micro-CT analysis revealed that LIPUS attenuated the alveolar bone resorption under pressure. In other studies, LIPUS promoted compensatory alveolar bone formation in rat OTM models after 14 days stimulation (Arai et al., 2020). LIPUS can effectively reduce orthodontically induced root resorption in rats (Wang et al., 2012) and beagle dogs (Wang et al., 2012; Al-Daghreer et al., 2014). It is noteworthy that we observed that LIPUS stimulation alone does not exert any influence on the osteogenic differentiation of PDLCs. Lucas et al. applied LIPUS with an average intensity of 100 mW/cm^2^ to mesenchymal precursors and found no significant changes in cell proliferation, morphology, and cytoskeleton (Garcia Aznar et al., 2021). Ultrasound alone has no effect on the neuronal differentiation of NSCs (Zhang et al., 2023b). Gao et al. have also reported that LIPUS with an intensity of 750 mW/cm^2^ has no significant effect on the expression of Piezo1 in PDLSCs (Gao et al., 2017). Therefore, we are of the opinion that the activation of Piezo1 may be the prerequisite for LIPUS to promote the osteogenesis of force-treated PDLCs in our study.

In this study, mechanical force increased the expression of Piezo1 in PDLCs. This observation aligns with previous research findings (Jin et al., 2015; Shen et al., 2020; Jiang et al., 2021; Du and Yang, 2023). Piezo1 is necessary for bone formation and bone metabolism (Li et al., 2019; Sun et al., 2019). In mesenchymal or osteoblast progenitor cells, loss of Piezo1 inhibits osteoblast differentiation and increases bone resorption, leading to multiple spontaneous bone fractures in newborn mice (Wang et al., 2020). The activation of the Piezo1 channel is essential for facilitating new alveolar bone formation on the tension side (Jiang et al., 2021). Nevertheless, our study suggested that compressive force/Yoda1 inhibits the osteogenic differentiation of PDLCs via upregulating Piezo1. Conversely, LIPUS restored the osteogenesis of PDLCs by downregulating Piezo1. Activation of Piezo1 in PDLCs under pressure promotes the expression of RANKL/OPG (Schröder et al., 2023). RANKL/OPG is a key factor influencing local bone formation and resorption activities, with decreased OPG expression leading to reduced bone-forming ability (Du and Yang, 2023). We administered the Piezo1 inhibitor GsMTx4 to rats. Similarly, we used siPiezo1 to knockdown the Piezo1 of hPDLSCs *in vitro*. GsMTx4 alleviated bone resorption induced by orthodontic force in rats and increased the number of COL1A1-and OCN-positive cells in the PDL on the compressive side. But there was no significant difference in the number of COL1A1-and OCN-positive cells between the Force + GsMTX4 group and the Force + GsMTX4+LIPUS group. *In vitro*, the osteogenic potential of siPiezo1-pretreated hPDLCs (Force + siPiezo1 and Force + siPiezo1+LIPUS groups) was stronger than that of force-treated hPDLCs. However, LIPUS did not promote the osteogenic ability of force-treated hPDLCs after piezo1 knockdown (Force + siPiezo1 group versus Force + siPiezo1+LIPUS group). Therefore, we are of the opinion that the effect of LIPUS was partially blocked by the inhibition of Piezo1. On the contrary, we used Yoda1 to activate Piezo1, resulting in the inhibition of osteogenesis in PDLCs. Similar to force-treated PDLCs, LIPUS promoted the osteogenic ability of Yoda1-treated PLDCs. Similarly, activation of Piezo1 ion channels in human odontoblasts by mechanical force/Yoda1 inhibits their mineralization capacity. Knockdown of Piezo1 in human odontoblasts enhances their mineralization capacity (Matsunaga et al., 2021). Additionally, Zhang et al. used a combination of 2D nanomaterials and ultrasound to downregulate Piezo1 and promote neurogenesis in the treatment of spinal injury (Zhang et al., 2023b). Consequently, we conclude that Piezo1 exhibits a bidirectional effect in tissue repair and regeneration under different conditions. In the part of Piezo1 function activation and inhibition experiments, we did not establish separate controls based on the clear effects observed in Figures 1, 2. These figures demonstrated changes in OTM and periodontal tissue remodeling after force and LIPUS application. To minimize animal usage in subsequent experiments, we opted not to include additional control groups. Instead, we compared each experimental group directly to the Force group. While this approach allowed for a focused examination of Piezo1 modulation effects, it is important to note that the absence of specific control groups may limit our understanding of the underlying mechanisms. Future studies could consider incorporating additional controls to address this limitation.

To summarize, we are of the opinion that LIPUS attenuated alveolar bone resorption under pressure by promoting the osteogenesis of force-treated PDLCs through the downregulation of Piezo1. However, this study has not yet fully explained the mechanism underlying how LIPUS affects the osteoclastic differentiation under force application, which needs further investigation. In our study, the injection of GsMTx4 resulted in a reduction in OTM in rats, which aligns with the findings from previous research (Jiang et al., 2021; Xu et al., 2022). Jiang et al. demonstrated that GsMTx4 injection led to a decrease in osteoclasts at the alveolar bone on the tension side, resulting in a reduced tooth movement distance. GsMTx4 is not specific to Piezo1 and may also inhibit other ion channels (Gnanasambandam et al., 2017); therefore, we would also conduct additional research using Piezo1-knockout mice to confirm the functional impact of Piezo1 in the application of LIPUS during OTM.

## 5 Conclusion

**FIGURE 9:**
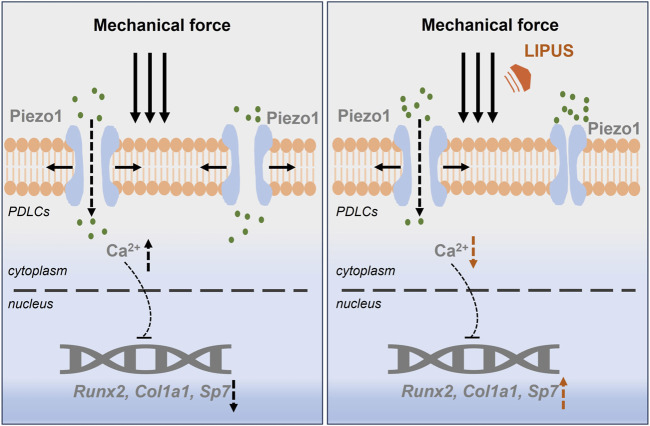
LIPUS promotes the osteogenesis of force-treated PDLCs via downregulating Piezo1.

In summary, LIPUS promotes the osteogenesis of force-treated PDLCs via downregulating Piezo1, which offers new insight into the regulation of bone homeostasis during orthodontic treatment (Figure 9).

## Data Availability

The raw data supporting the conclusion of this article will be made available by the authors, without undue reservation.
