# Supplementary figures and images for: Low-intensity pulsed ultrasound promotes the osteogenesis of mechanical force-treated periodontal ligament cells via Piezo1
Source: Front Bioeng Biotechnol. 2024 Apr 17;12:1347406. doi: 10.3389/fbioe.2024.1347406 (PMC11061374; doi:10.3389/fbioe.2024.1347406)

Figure3

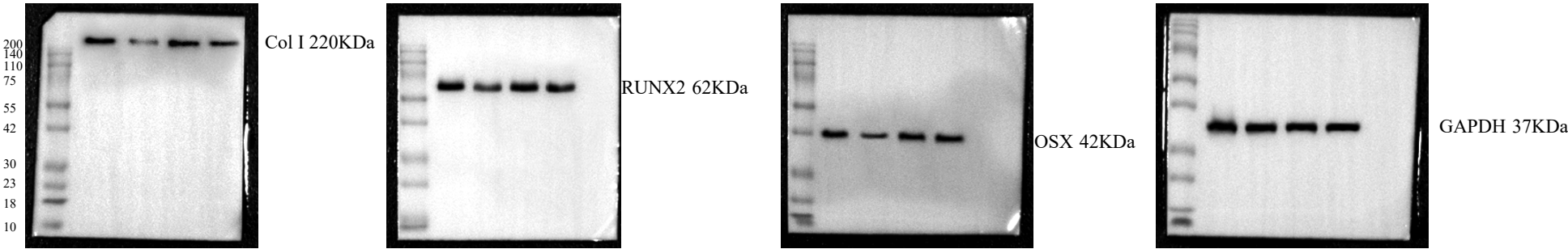

Figure4

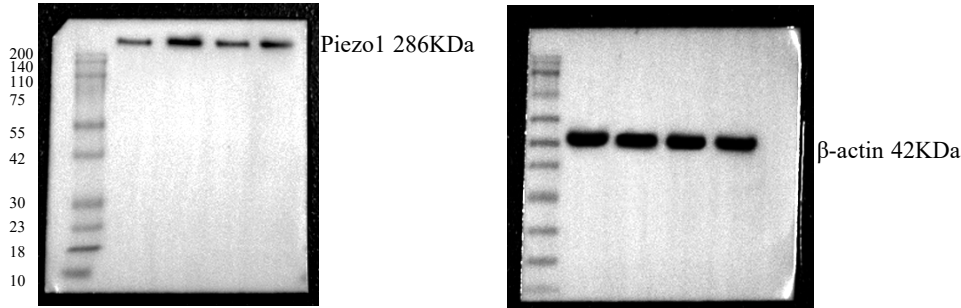

Figure5

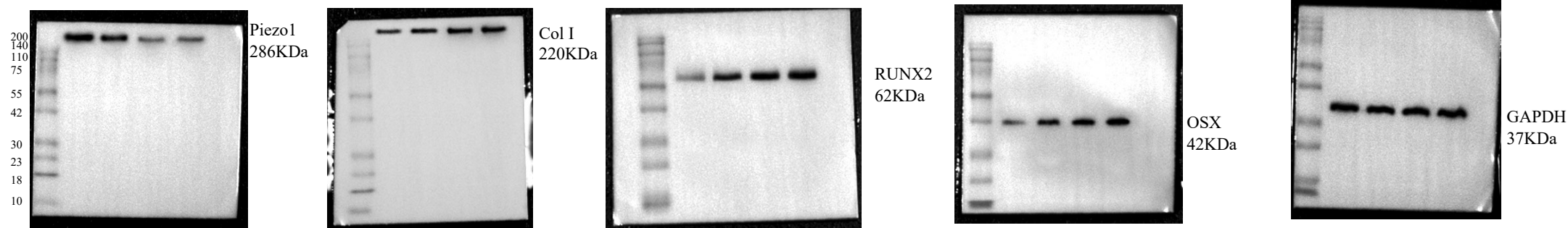

Figure7

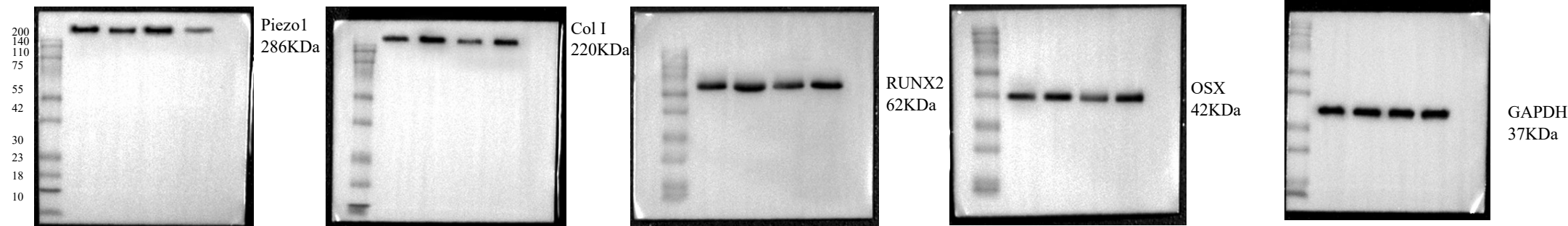

Supplement: Supplementary file 1 [file DataSheet2.pdf]
