# Supplementary material for: Low-intensity pulsed ultrasound promotes the osteogenesis of mechanical force-treated periodontal ligament cells via Piezo1
Source: Front Bioeng Biotechnol. 2024 Apr 17;12:1347406. doi: 10.3389/fbioe.2024.1347406 (PMC11061374; doi:10.3389/fbioe.2024.1347406)
Supplement: Supplementary file 2 [file DataSheet1.docx]

Supplementary Material

**Table S1 Nucleotide sequence of primers used in qPCR.**

| Gene | Forward primer | Reverse primer |
| --- | --- | --- |
| For human periodontal ligament cells (hPDLCs) |  |  |
| glyceraldehyde-3-phosphate dehydrogenase *(GAPDH)* | TCATTGACCTCAACTACATG | TCGCTCCTGGAAGATGGTGAT |
| runt-related transcription factor 2 *(RUNX2)* | TGGTTACTGTCATGGCGGGTA | TCTCAGATCGTTGAACCTTGCTA |
| collagen type I alpha 1 (*COL1A1)* | GTGCGATGACGTGATCTGTGA | CGGTGGTTTCTTGGTCGGT |
| Sp7 Transcription Factor *(SP7)* | ATAGTGGGCAGCTAGAAGGGAGTG | ATTAGGGCAGTCGCAGGAGGAG |
| For RAW264.7 |  |  |
| *Gapdh* | TTGCAGTGGCAAAGTGGAGA | GATGGGCTTCCCGTTGATGA |
| acid phosphatase 5, tartrate resistant (*Acp5)* | ACACAGTGATGCTGTGTGGCAACTC | CCAGAGGCTTCCACATATATGATGG |
| matrix metalloproteinase 9 (*Mmp9*) | CTGGACAGCCAGACACTAAAG | CTCGCGGCAAGTCTTCAGAG |
| cathepain K *(Ctsk)* | GAAGAAGACTCACCAGAAGCAG | TCCAGGTTATGGGCAGAGATT |
| nuclear factor 1 of activated T cells *(Nfatc1)* | CAGTGTGACCGAAGATACCTGG | TCGAGACTTGATAGGGACCCC |

**Supplemental Figure 1**


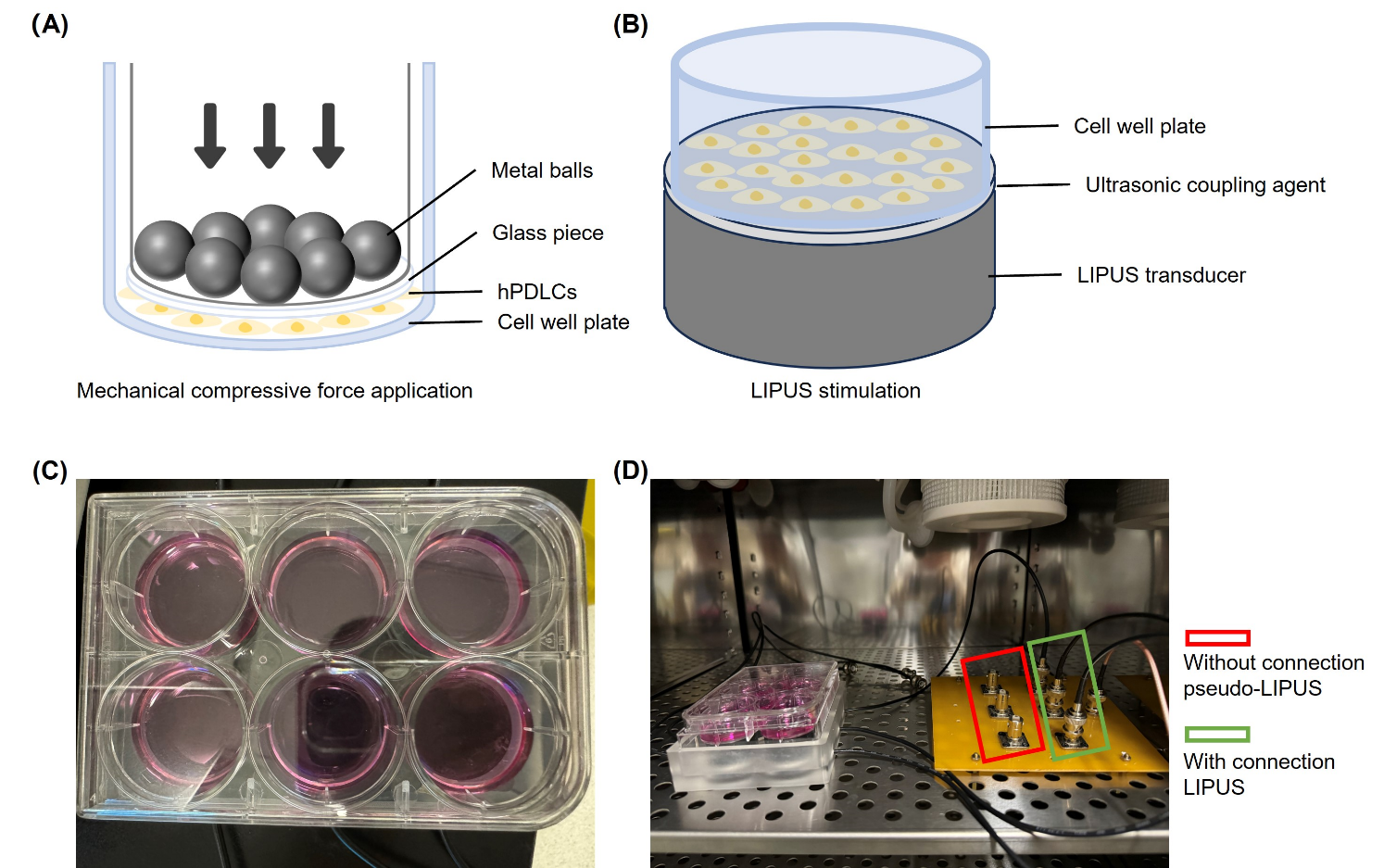


**Figure. S1** Application of mechanical compressive force and LIPUS to hPDLCs. **(A)** The schematic representation of applying mechanical compressive force to hPDLCs. **(B)** The schematic diagram illustrating the application of LIPUS stimulation to hPDLCs. **(C)** The photograph depicting the positioning relationship between the well plate and the LIPUS transducers. **(D)** The photograph of LIPUS and pseudo-LIPUS administration in cell culture at 37°C.

**Supplemental Figure 2**


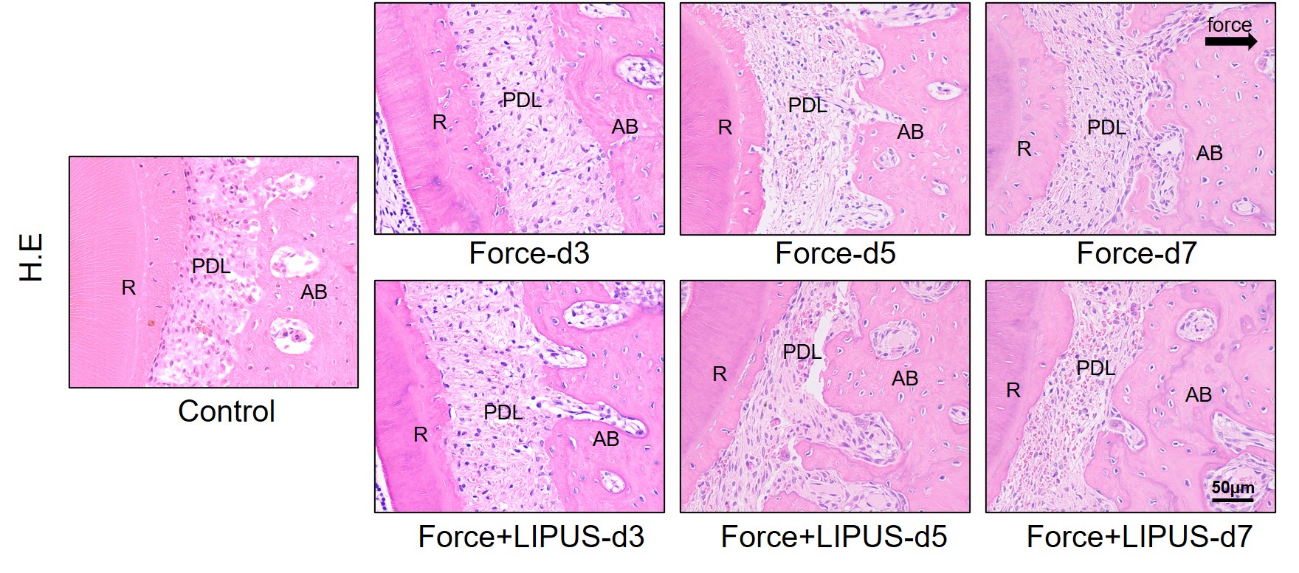


**Figure. S2** Representative hematoxylin and eosin (H&E) staining images of the distobuccal root's compression side. The long black arrow indicates the force direction. The H&E staining indicates that compared to the control group, the fiber arrangement is disordered in the Force group and the Force+LIPUS group. Scale bar: 50 μm (n=3).

**Supplemental Figure 3**


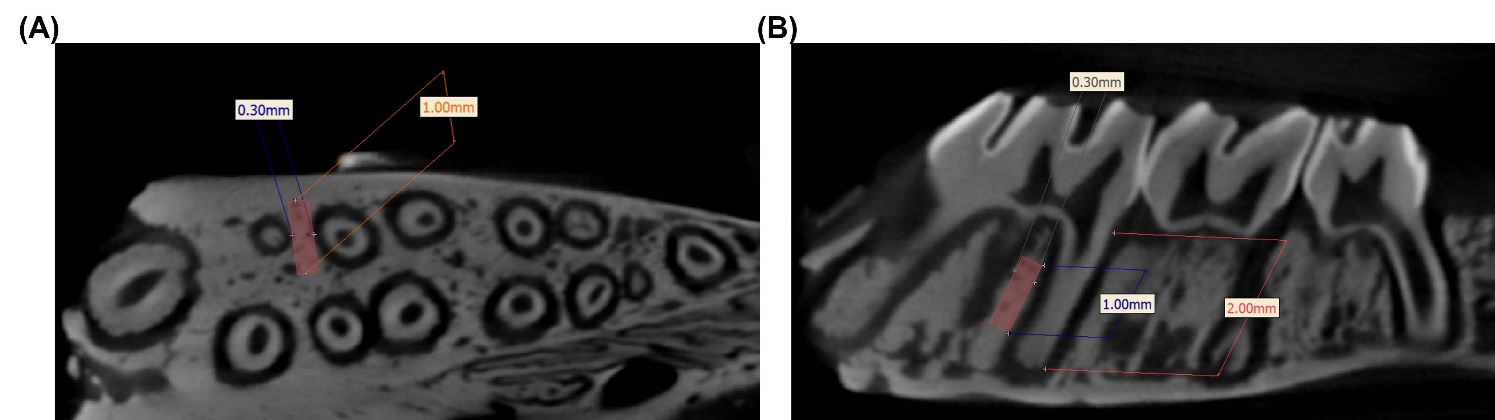


**Figure. S3** A square-shaped region of interest (ROI) measuring 0.3*1*1 mm in volume was selected at the mesial aspect of the distobuccal root of the maxillary first molar after three-dimensional reconstruction. **(A)** Cross-sectional with ROI delineation. **(B)** Coronal views with ROI delineation.

**Supplemental Figure 4**


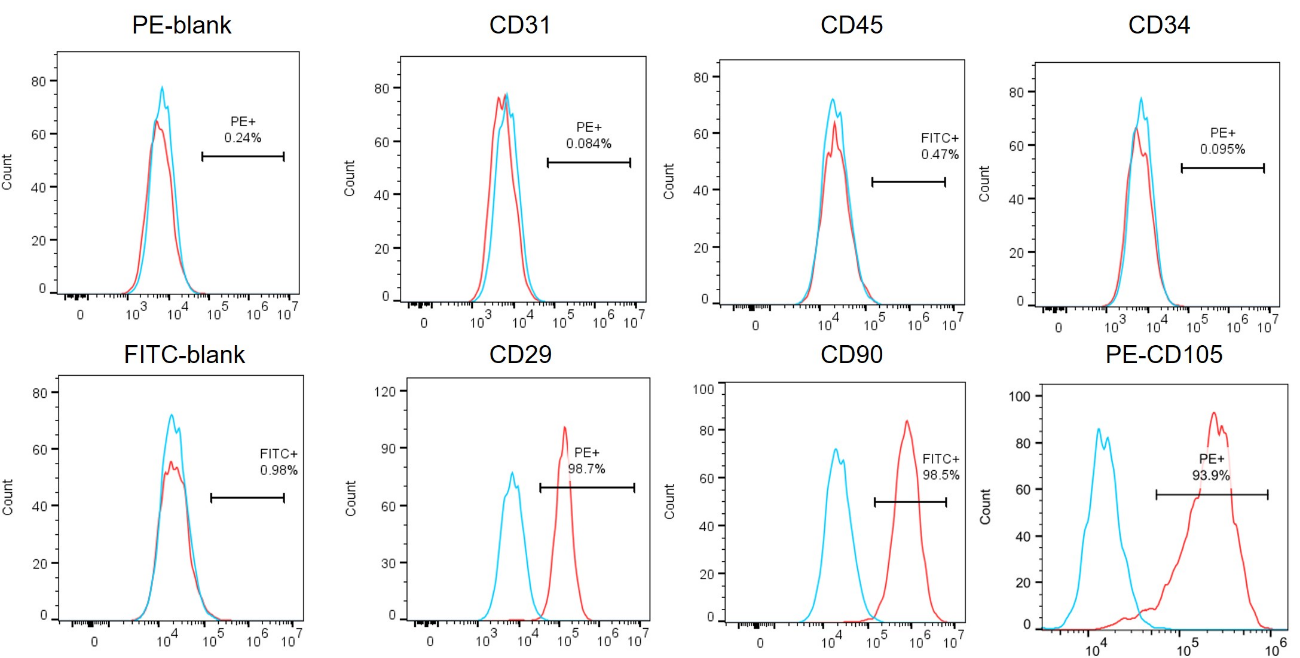


**Figure. S4** Expression of cell surface markers CD31, CD45, CD34, CD29, CD90 and CD105 in human periodontal ligament cells (hPDLCs) were detected by flow cytometry. Surface markers CD31, CD45, and CD34 were negative, while CD29, CD90 and CD105 were positive.

**Supplemental Figure 5**


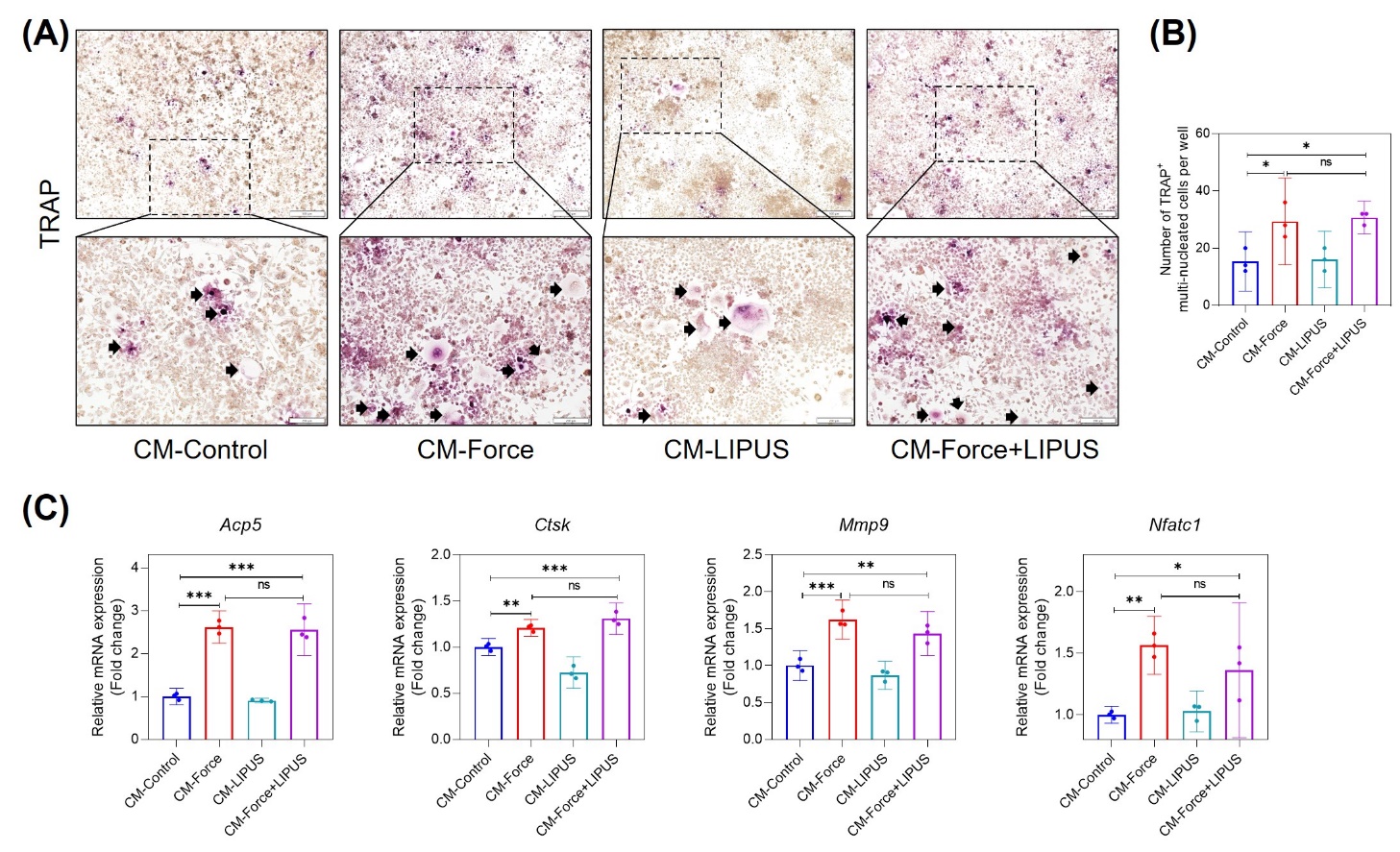


**Figure. S5** The effect of conditional medium of hPDLCs after different treatments on osteoclast differentiation of RAW264.7 **(A-B)** Representative TRAP staining images of RAW264.7 treated by conditional medium. The short black arrows indicate TRAP-positive multinucleated osteoclasts. Semi-quantitative analysis demonstrates Force and Force+LIPUS treatment promoted the osteoclast differentiation. However, there is no significant difference in the number of osteoclasts between the two groups. Scale bar: 500 μm (up) and 200 μm (down) (n=3). **(C)** Expression changes of *Acp5, Mmp9, Ctsk* and *Nfatc1* at mRNA levels in RAW264.7 are determined by qPCR. Data represent as mean with 95% confidence interval. *P < 0.05; **P < 0.01; ***P < 0.001; ns, not significant (P＞0.05). All experiments were repeated three times. CM, conditional medium; LIPUS, low-intensity pulsed ultrasound.

**Supplemental Figure 6**


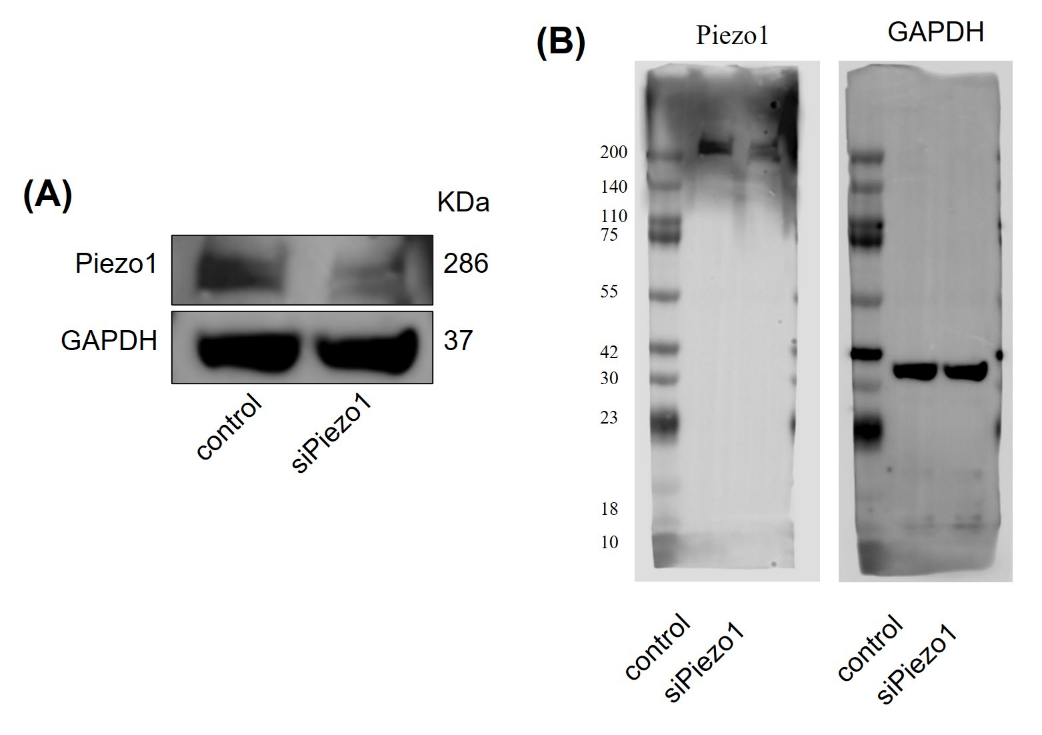


**Figure. S6** siPiezo1 knocked down Piezo1 expression in hPDLCs. **(A)** Western blot results show that changes in the protein levels of Piezo1 in hPDLCs after siPiezo1 transfection. **(B)** The uncropped Western blot membrane.

**Supplemental Figure 7**


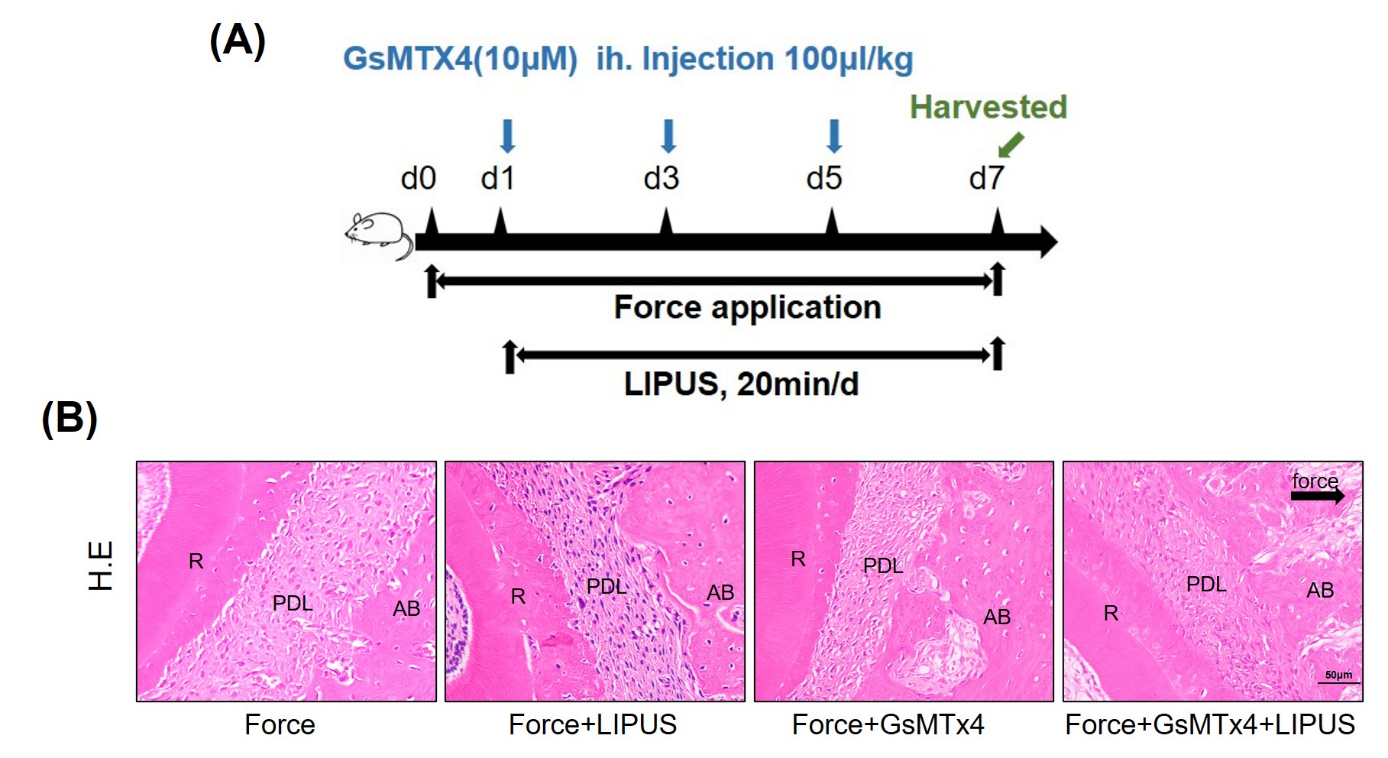


**Figure. S7** Schedule diagram of the Piezo1 inhibition experiment.

**Supplemental Figure 8**


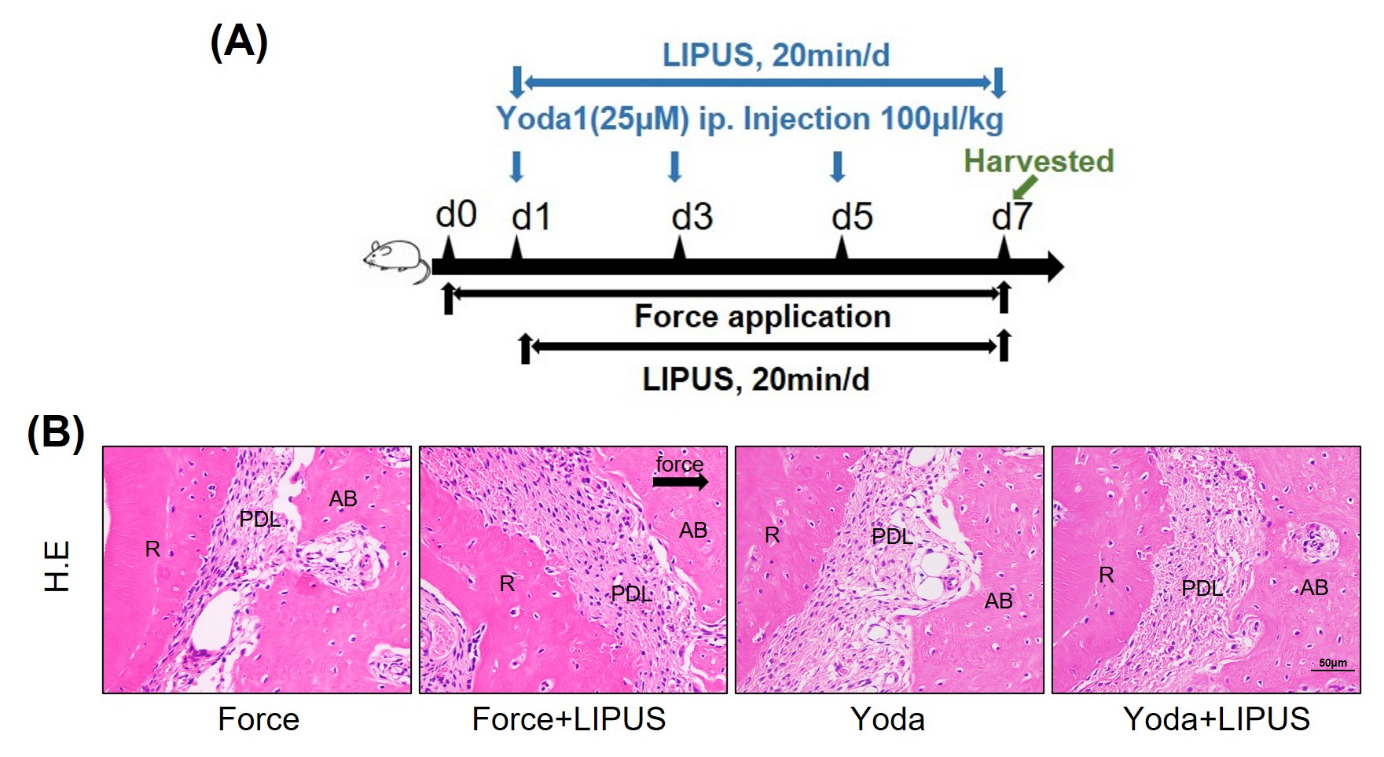


**Figure. S8** Schedule diagram of the Piezo1 activation experiment.
